# Environmental Gap Analysis to Prioritize Conservation Efforts in Eastern Africa

**DOI:** 10.1371/journal.pone.0121444

**Published:** 2015-04-09

**Authors:** Paulo van Breugel, Roeland Kindt, Jens-Peter Barnekow Lillesø, Michiel van Breugel

**Affiliations:** 1 Forest, Nature and Biomass, Department of Geosciences and Natural Resource Management, University of Copenhagen, Rolighedsvej 23, Frederiksberg C, DK-1958, Denmark; 2 World Agroforestry Centre, P.O. Box 30677–00100, Nairobi, Kenya; 3 Yale-NUS College, Department of Biological Sciences, National University of Singapore, Singapore, 6 College Avenue East, #06-01D, Singapore, 138614; 4 Center for Tropical Forest Science, Smithsonian Tropical Research Institute, Ciudad de Panamá, República de Panamáa; 5 Department of Biological Sciences, National University of Singapore, 14 Science Drive 4, Singapore, Singapore, 117543; Instituto de Pesquisas Ecológicas, BRAZIL

## Abstract

Countries in eastern Africa have set aside significant proportions of their land for protection. But are these areas representative of the diverse range of species and habitats found in the region? And do conservation efforts include areas where the state of biodiversity is likely to deteriorate without further interventions? Various studies have addressed these questions at global and continental scales. However, meaningful conservation decisions are required at finer geographical scales. To operate more effectively at the national level, finer scale baseline data on species and on higher levels of biological organization such as the eco-regions are required, among other factors. Here we adopted a recently developed high-resolution potential natural vegetation (PNV) map for eastern Africa as a baseline to more effectively identify conservation priorities. We examined how well different potential natural vegetations (PNVs) are represented in the protected area (PA) network of eastern Africa and used a multivariate environmental similarity index to evaluate biases in PA versus PNV coverage. We additionally overlaid data of anthropogenic factors that potentially influence the natural vegetation to assess the level of threat to different PNVs. Our results indicate substantial differences in the conservation status of PNVs. In addition, particular PNVs in which biodiversity protection and ecological functions are at risk due to human influences are revealed. The data and approach presented here provide a step forward in developing more transparent and better informed translation from global priorities to regional or national implementation in eastern Africa, and are valid for other geographic regions.

## Introduction

The state of biodiversity is continuing to deteriorate, with species and ecosystems increasingly threatened by the human appropriation of earth's natural resources [1–3]. This has led to a clear increase in policy and management responses. This includes a considerable growth in protected areas [4], which are recognized as an important global strategy for conservation. As a result, in the eastern African countries of Kenya, Malawi, Rwanda, Tanzania, Uganda and Zambia, a total of almost 27% of their land surface area has been assigned as PAs [4]. This proportion is well above the 17% target adopted as “target 11” of the Biological Diversity Strategic Plan for Biodiversity 2011–2020 [5].

Although PA coverage in eastern Africa is high, as in other regions the current framework of protected areas is the result of historical processes that have evolved based on various principles and objectives that do not necessarily comprise a logical whole. The earliest parks in Africa were established based on contemporaneous European ideals of the pristine wilderness [6] and on economic considerations [7]. But the rather ad hoc nature of assignment has resulted in a network of PAs that is not always ecologically representative, with a tendency to bias assignment to less accessible locations [8,9], or with low agricultural potential [10,11]. Only relatively recently has a wider view of biodiversity conservation prevailed in the assignment of PAs [12–14]., but even this view has not always resulted in better ecological representation [15].

An essential first step to improve PA networks is to identify gaps in coverage of biodiversity and of distinct environments [16]. Effective conservation planning should also consider the vulnerability of areas outside the PAs to biodiversity loss [17,18]. Various global prioritization schemes have been proposed in the last decades, based on a range of different criteria [19–22]. These may focus on biodiversity hotspots [23–29], or on well-preserved wilderness areas [30,31]. Clearly, detailed information on biodiversity in area of interest is desirable, but for the majority of species there are significant gaps in our knowledge regarding distributions, with information often limited to only a portion of natural ranges [32–35]. For a few (perhaps iconic) species better information is available, but this may not be representative of other flora and fauna [36–38]. Moreover, biodiversity indicators may not always represent well other environmental values [17], such as environmental or habitat diversity [39,40], environmental quality, ecosystem functions and intactness or rarity of ecosystems [41,42].

An alternative approach for conservation planning is to look at higher levels of biological organization such as biomes, eco-regions and habitat units [43–47]. This provides a framework for the identification of representative habitats and species assemblages at different scales [41,48,49], allowing a critical spatial linkage between global priority-setting efforts and site-based assessments [50]. Such an approach furthermore allows for a broader view beyond the protection of species to the conservation of a variety of landscapes, ecological interactions and ecosystem services. In the case of Africa, perhaps the most widely used map in this regard, is the terrestrial ecoregional map [45], based largely on Frank White’s well-known vegetation map of Africa [51]. This provides a good baseline for conservation planning at the continental level [41,48,52], but the limited scale of the map (1:5 million), the limited precision, and the high aggregation of vegetation units, makes it less suitable for use nationally.

In the study presented here we counter this limitation by using a detailed potential natural vegetation map recently constructed by some of the current authors for the eastern Africa region [53], based on historical national vegetation maps, literature sources and expert knowledge. In this map, a potential natural vegetation (PNV) is defined as the vegetation that would persist within a given area under environmental conditions, including those created by man, at the time the national vegetation maps were created [54]. While the scope of the information presented is similar to existing maps, ours is significantly more detailed and therefore more relevant for planning at the sub-regional and national level.

With our map as baseline, we wished to address here two questions important for effective conservation planning. First, how well does the current PA network in eastern Africa represent the range of PNVs present in the region? And, second, to what extent are different PNVs under threat due to human pressure that may lead to the degradation or even permanent loss of the current PNV. Addressing the second question involved estimating the level of human pressure and the proportion of each PNV that had been converted. Our approach, which involved combining information on levels of threats and representativeness to identify gaps in current PA networks, has been applied elsewhere [10,41,55,56], but until the current study not in eastern Africa. An additional feature of our study was to examine how well PAs represent environmental conditions within PNVs [57–59]. We discuss how priorities for the region based on our high-resolution baseline data and our methodologies fit into commonly accepted global conservation priorities.

## Methods

### Data availability

All data sets developed for this study are publicly available at http://vegetationmap4africa.org/applications. For third-party data sets, full references are given in the text.

### Study area

Our study covers the African nations of Kenya, Malawi, Rwanda, Tanzania, Uganda and Zambia. Together these countries of eastern Africa cover an area of 2,659,807 km^2^. The region harbors a diverse range of ecosystems, including the dry plains of northern Kenya and the rainforests and alpine moorlands of the two highest mountains of Africa (Mount Kenya in Kenya, Mount Kilimanjaro in Tanzania). Annual rainfall varies from less than 400 mm in northern Kenya to more than 1500 mm around Lake Victoria and in the higher mountain ranges. With an urban population of 22% [60], the region is one of the least urbanized in the world, although in the coming decades the percentage urbanization is expected to increase to more closely match that of Africa as a whole (currently 39%) [61].

### Baseline potential natural vegetation map

As a baseline representation of the main woody plant communities in eastern Africa, we used a high resolution PNV map for eastern Africa recently developed by some of the current authors and our partners [62]. The map is a harmonized composite of national maps that were developed based on botanical field surveys undertaken mainly between 1950 and 1970 [63]. The resulting map was adapted for our regional analyses by reclassifying the PNVs in some of the countries only [53]. The map, shown in S1 Fig and available for download at http://vegetationmap4africa.org/conservation, consists of 50 PNVs that can be categorized under six major headings. Eleven PNVs are categorized under forest, 13 under open forest and woodland, 15 types under bushlands, thickets and wooded grasslands, 5 under highland vegetation, 2 under arid zones and 4 types under grassland and herbaceous vegetation (Table 1).

**Table 1:** Names and codes of potential natural vegetations in eastern Africa.

****Category****	****PNV****	****Code****
**Forest PNVs**	Afromontane rain forest	Fa
	Afromontane undifferentiated forest	Fb
	Single-dominant *Hagenia abyssinica* forest	Fd
	Afromontane moist transitional forest	Fe
	Lake Victoria transitional rain forest	Ff
	Zanzibar-Inhambane transitional rain forest	Fg
	Afromontane dry transitional forest	Fh
	Lake Victoria drier peripheral semi-evergreen Guineo-Congolian rain forest	Fi
	Zambezian dry evergreen forest	Fm
	Zambezian dry deciduous forest and scrub forest	Fn
	Zanzibar-Inhambane lowland rain forest	Fo
**Woodland PNVs**	Coastal mosaic	CM
	Mangrove	M
	Dry *Combretum* wooded grassland	Wcd
	Moist *Combretum* wooded grassland	Wcm
	Zambezian Kalahari woodland	Wk
	Drier miombo woodland	Wmd
	Miombo woodland on hills and rocky outcrops	Wmr
	Wetter miombo woodland	Wmw
	North Zambezian undifferentiated woodland	Wn
	Mopane woodland and scrub woodland	Wo
	*Vitex*-*Phyllanthus*-*Sapium*-*Terminalia* and *Terminalia glaucescens* woodland	Wv
	Zambezian chipya woodland	Wy
	Transitional zone of drier miombo woodland and North Zambezian Undifferentiated woodland	Wmd/Wn
**Bushland, thickets and wooded grassland PNVs**	Somalia-Masai *Acacia*-*Commiphora* deciduous bushland and thicket	Bd
	*Acacia*-*Commiphora* stunted bushland	Bds
	*Acacia*-*Commiphora* deciduous wooded grassland	Bdw
	Catena of *Acacia*-*Commiphora* deciduous wooded grassland, *Combretum* wooded grassland and edaphic grassland on drainage-impeded or seasonally flooded soils	Bdw/Wc/g
	Evergreen and semi-evergreen bushland and thicket	Be
	Itigi thicket	bi
	Lowland bamboo	L
	Palm wooded grassland	P
	Bush groups, typically around termitaria, within grassy drainage zones	T/g
	*Vitellaria* (synonym: *Butyrospermum*) wooded grassland	Wb
	Edaphic wooded grassland on drainage-impeded or seasonally flooded soils	wd
	Upland *Acacia* wooded grassland	We
	Zambezian Kalahari woodlands within edaphic grassland on drainage-impeded or seasonally flooded soils	Wk/g
	Transitional zone of drier miombo woodland and Somalia-Masai *Acacia*-*Commiphora* deciduous bushland and thicket	Wmd/Bd
	Catena of North Zambezian Undifferentiated woodland and edaphic grassland on drainage-impeded or seasonally flooded soils	Wn/g
**Grassland PNVs**	Climatic grasslands	G
	Edaphic grassland on drainage-impeded, seasonally flooded soils or freshwater swamp	g/X
	Afromontane forest—grasslands mosaic	gm/F
	Edaphic grassland on volcanic soils	gv
**Highland PNVs**	afroalpine vegetation	A
	Afromontane desert	Ad
	afromontane bamboo	B
	Montane Ericaceous belt	E
	Mosaic of Montane Ericaceous belt and Single-dominant *Widdringtonia whytei* forest	E/Fc
**Arid zone PNVs**	Desert	D
	Somalia-Masai semi-desert grassland and shrubland	S

### Geographical coverage of the potential natural vegetation in relation to protected areas

For each PNV, we calculated the percent area covered by PAs, which we will henceforth refer to as the geographic coverage index (GC), using the World Database on Protected Areas (WDPA) [4]. The WDPA places PAs into seven different International Union for Conservation of Nature (IUCN) management categories, based on their principle management objectives [64]. Five of the seven categories are found in the eastern Africa region, namely; Ib, Wilderness Area; II, National Park; III, Natural Monument or Feature; IV, Habitat/Species Management Area; and VI, Protected Area with Sustainable Use of Natural Resources. We reclassified these five categories into two groups, PA1 and PA2. PA1 is composed of the IUCN categories Ib, II, III and IV, all of which are explicitly designated for biodiversity or landscape protection. PA2 is composed of IUCN category VI, which is designated for both protection and sustainable use objectives. In addition, PA2 includes unclassified (according to IUCN terminology) PAs, such as different types of national or community forest reserves and areas that have a focus on wildlife or game management. We assume that the management of the PA2 category is less likely to be focussed on the conservation of PNVs. It should be noted that this does not imply any assumptions on the effectiveness of the management in these different categories (see discussion).

In our compilation, we only considered nationally recognized PAs as these represent areas were the respective national or local governments have a legally binding commitment to protect or use the land and its natural resources in a sustainable way. Where PAs of different IUCN categories overlapped, we assigned the highest IUCN classification ranking to the overlapped areas. Excluded from analysis were areas proposed for protection but not yet assigned protected area status, areas that were represented by point data only and marine locations.

### Environmental representation

To identify possible biases in the distribution of PAs along environmental gradients within the PNVs, and to highlight for each PNV the parts with environmental conditions poorly represented within the PA network, we computed the multivariate environmental similarity index (MES). This index is akin to the environmental representativeness or distinctiveness [57,65] and measures how similar a point (*n*) is to a set of reference points (*p*) in terms of a set of predictor variables (*V*_*1*_, *V*_*2*_…*V*_*i*_) [66]. Further explanation of the calculation of MES in the current study is given in S1 Appendix. As predictor variables, we used the aridity index [67], a 90 m digital elevation [68], the terrain wetness index (twi, calculated using the r.topidx function in GRASS GIS [69]), the river density (based on the EON river database [70]) and 19 bioclimatic variables, listed in S1 Table. All layers were resampled to a 900 m resolution for further analysis.

We created two MES surfaces for each PNV. MES1 represents how similar environmental conditions in a given location are to the overall conditions for the PNV. MES2 represents how similar the conditions in a given location are to the conditions found in the PAs of the PNV. Based on MES1, we compared the distribution of values in the PAs (MES1_PA_) to the MES values in the entire PNV (MES1_PNV_). We used the absolute difference of the median of MES1_PA_ and MES1_pnv_ divided by the median absolute deviation to measure whether environmental conditions in the PA are biased towards more common or less common environmental conditions for the PNV. We will henceforth refer to this statistic as the environmental bias (EB). For a more detailed explanation, see S1 Appendix.

### Analysis of threats

To identify the most threatened PNVs, and within each PNV the parts where the anthropogenic pressure is likely to lead to the degradation or conversion of the natural vegetation, we combined information on land conversion with data on major potential drivers of vegetation cover changes. This approach has been used by the Global Methodology for Mapping Human Impacts on the Biosphere project [71] and the Global Human Footprint project [72], amongst other initiatives. We first computed the proportion of the PNVs where all natural vegetation was cleared. For the remaining areas, we calculated the human influence (HI). The human influence refers to the relative anthropogenic pressure on the natural vegetation, and was estimated based on four different human factors as described in the subsequent sections.

**Fig 1:**
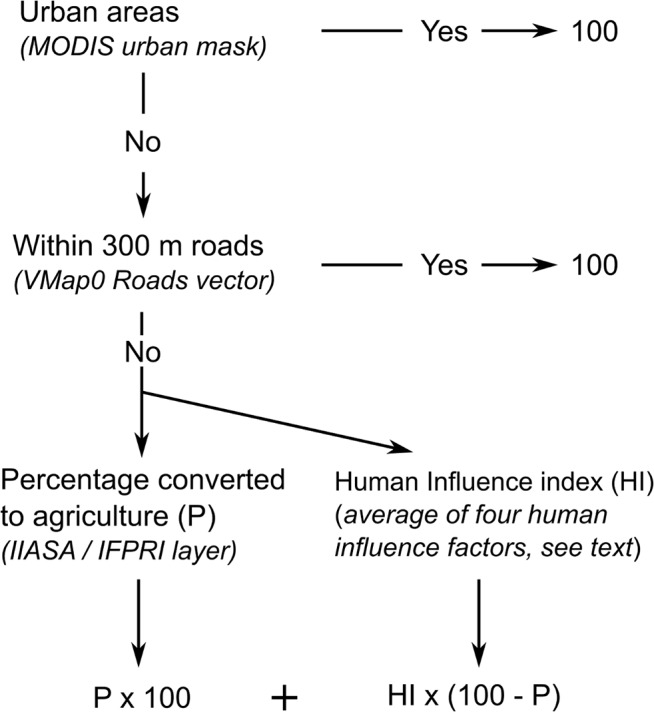
Framework to compute the human influence index.

This two-step approach (Fig 1) differs from the HI index developed by Sanderson et al. [72], who used land transformation as one of the threat layers that were summed to obtain the HI score. Our index thus provides an estimate of the loss and potential degradation of the PNV cover, avoiding the assumption that the biodiversity value of agricultural or urban areas is equal to zero. An outline of the two steps is provided below, while a more detailed explanation is given in S2 Appendix.

For each PNV, the percentage of land where the natural vegetation was cleared, henceforth refered to as the conversion score, was estimated based on three maps, namely: the global cropland map [73,74] (available from http://beta-hybrid.geo-wiki.org/), the MODIS 2009 urban areas mask [75] (available from http://sage.wisc.edu/people/schneider/research/data.html) and the VMap0 Roads vector layer (available from http://gis-lab.info/qa/vmap0-eng.html). We assigned a conversion score of 100 to areas converted to urban areas or roads. The conversion score for croplands was equal to the percentage of land identified as croplands on the global cropland map. Conversion in the context above refers to the partial or complete replacement of the PNV cover with other land cover types (urban areas, croplands, secondary vegetation).

For non-converted lands we computed the relative human influence [72]. As indicators, we used: the relative change in the vegetation physiognomy between potential and actual vegetation cover; human population density; travel distance to high population density areas; and livestock grazing pressure. A short description of each of these is given below, while a more detailed account is provided in S2 Appendix. Each indicator received a score of between 0 and 100, representing a scale of no (0) to maximum (100) influence. We took the arithmetic mean of these four scores and multiplied by the percentage of non-converted land. This value was added to the conversion score to obtain the final HI score.

#### Relative change in the vegetation physiognomy

We compared the physiognomy of the PNV map with the physiognomy of four land use cover (LUC) maps: the Globcover regional land use cover map version 2.2 [76]; the Global Land Cover 2000 (GLC2000) map for Africa, version 3 [77]; the MODIS Land Cover data, Land Cover Type 1, IGBP global vegetation classification scheme for 2005; and the MODIS Land Cover data, Land Cover Type 1, University of Maryland (UMD) scheme [78,79]. Values of 25, 50, 75 and 100 were assigned when the physiognomy of a LUC map was respectively 1, 2, 3 or 4 steps below the physiognomy of the PNV map, in the sequence: (1) forest vegetation; (2) open forest or woodland vegetation; (3) bushland, thicket and wooded grassland; (4) grasslands and herbaceous vegetation; (5) stunted bushland; and (6) semi-desert. The arithmetic mean score over the four LUC maps was used as an indicator of degradation of the vegetation cover (VTI).

#### Human population density index

The number of people in a given area is frequently cited as an important cause of declines in species and ecosystems [80]. How human influences scale with human population density is, however, largely unknown [72], as it will depend on a combination of factors including the type of land use, the vulnerability of the vegetation and soils to the different human activities, and specific requirements of particular plant and animal species. The absence of hard information necessitates the use of simple assumptions. For their mapping of wilderness areas, for example, Mittermeier et al. [30] excluded all areas with a population density of 5 people / km^2^ or above, while Sanderson et al [72] assumed that with 10 persons or more / km^2^, there is a direct relationship between human population density and impact. Gorenflo [81] found that biodiversity tends to decline at population densities of more than 10 people / km^2^. Kruska et al. [82] distinguished between rangelands (< 20 people per km^2^) and higher impact mixed farming systems (> 20 people / km^2^). We assumed that in the latter systems the natural vegetation has been cleared, whereas in the former systems the human impact on the natural vegetation was assumed to be related to the human population density. Consequently, areas with population densities larger than 20 / km^2^ were given a HI score of 100, while HI scores were calculated to increase linearly from 0 to 100 between 0 and 20 persons / km^2^. Human population densities were derived from the Afripop data base [83] (available at http://www.worldpop.org.uk/).

Travel distance to high population density areas. This distance, expressed as a travel time, to the nearest high population density area, was estimated using the method proposed by Nelson [84], except we excluded rivers and railways as means of transport, and considered rivers as barriers to movement. High population density areas were defined as those with a population density > 1000 persons / km^2^ (Afripop data base; [83]), or those marked as settlements on the VMap0 Populated Place Polygon Reference map. A linearly increasing score from 0 to 100 was assigned for travel times between 6 and 0 hours, and a score of 0 to all areas further than 6 hours away. We henceforth refer to this variable as the accessibility index (AI).

#### Livestock grazing pressure index

Livestock grazing is a major livelihood strategy in large parts of the region and can have a significant impact on natural vegetation. The main livestock species in east Africa in the (semi-)natural areas are cattle, goats and sheep, while especially in the drier regions there are also considerable numbers of camels, donkeys, and horses [85–87]. We used the cattle, goat, and sheep density layers from FAO [88], which provides estimates corrected for unsuitability and adjusted to match FAOSTAT (URL: http://faostat.fao.org) totals for the year 2005. Data for other species not available, so are estimations of the total livestock densities are likely to be too low. We used these layers to compute the livestock pressure index (LPI) as an indicator of the pressure exerted by livestock on natural vegetation. The LPI was defined as 1—the ratio of feed requirement and availability multiplied by 100, with values ≥ 1 set at 100. Details on how feed requirements and availability were estimated are provided in S2 Appendix.

### Conservation risk analysis and identification of priority areas for conservation

We assumed that where human influence is high and the level of protection is low, the risks of loss of biodiversity and ecological dysfunction will be greater. To identify PNVs that are most at risk, which we will henceforth term crisis PNVs, sensu Hoekstra et al. [55], we calculated for each PNV the ratio of the human influence score (HI_pnv_) and the percent area protected. Below we refer to this ratio as the conservation risk index (CRI).

All areas with a HI > 50 and a CRI > 10 were classified as critically endangered (CR). Areas with a HI > 40 and CRI > 4 were classified as endangered (EN) and areas with HI > 20 and CRI > 2 as vulnerable (VU). This follows the terminology and approach suggested by Hoekstra et al. [55], but using different CRI thresholds to better account for differences between PNVs in this particular region.

## Results

### Geographic representation of potential natural vegetation in the protected areas network

**Fig 2:**
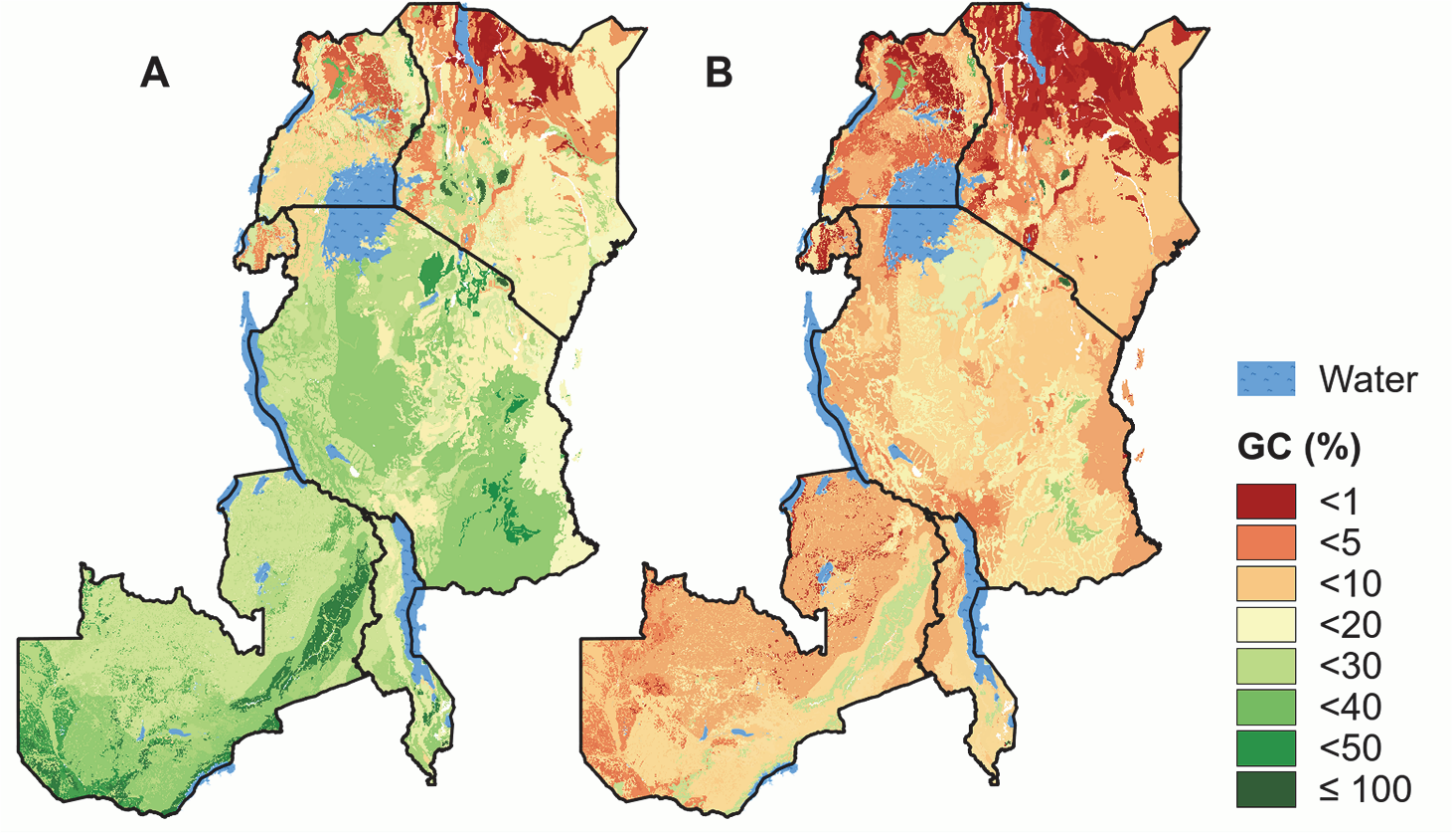
Geographic coverage of the potential natural vegetations. A) The percent area protected of potential natural vegetation types by the protected areas network (GC). B) As A, but only considering the more strictly protected PAs of IUCN class Ib-IV.

On average the *highland PNVs* are the best protected (85% of the highland PNVs occur in PAs), followed by the PNVs in *grasslands vegetation* and *open forests and woodlands* (both 31%), *the forests* PNVs (24%), the *bushlands*, *thickets and wooded grasslands* PNVs (21%) and the *arid zone* PNVs (1.2%). Except in the last case, this proportion is well above the global average of 12.7% geographic coverage of terrestrial surface areas [4]. There are, however, large differences between individual PNVs (Fig 2 and S1 Table). Best covered within the PA network are the *Afromontane desert* (Ad) and the *mosaic of Montane Ericaceous belt and Single-dominant Widdringtonia whytei forest* (E/Fc) (both 100%). At the other extreme are the *Somalia-Masai semi-desert grasslands and shrublands* (S) and the *deserts* (D), with less than 2% of the area protected in both cases. Overall, 40% of the PAs are classified as PA1 (more strictly protected). This percentage varies however for individual PNVs, from 100% for *afromontane desert* (Ad), *Afroalpine* (A) and *deserts* (D) zones, to 0% for *Mangrove* (M) and the *mosaic of Montane Ericaceous belt and Single-dominant Widdringtonia whytei forest* (E/Fc) zones.

The correlation between the total area of a PNV and the geographic coverage (GC) is weak and not significant, whether considering all PAs (r = -0.2, n.s.) or those placed into category PA1 (r = -0.2, n.s.). The best covered PNVs are the relatively restricted highland vegetation types, but other small PNVs, including *desert* (D) and *lowland bamboo* (L), are poorly represented in the PA network. Conversely, four of the five largest PNVs have an above average percentage within the PA network.

There are clear differences between countries in the geographic coverage of PNVs (Fig 2 and S1 Table) that cannot be entirely explained by human influence (S2 Fig). In Rwanda, Kenya, Malawi and Uganda respectively 10, 12, 14 and 15% of the terrestrial surface area is protected. In contrast, the percentage of protected land in Tanzania and Zambia is 30 and 35%, respectively. These differences are reflected in how different PNVs are covered. For example, the geographic coverage of PNVs that occur in both Kenya and Uganda is generally higher in Uganda, while the percent area of the coastal mosaic that is protected in Tanzania (23%) is considerably higher than in Kenya (13%). That most of the miombo woodlands and related vegetation types are well represented in PAs is directly linked to the relatively high percentage coverage by PAs in Zambia and Tanzania, where most of these vegetation types are found. These patterns differ when considering PA1 only. For example, the *Somalia-Masai Acacia-Commiphora deciduous bushland and thicket* (Bd) in the north is underrepresented in the PA network, but from what is protected, a relative large portion (40%) is of category PA1. In contrast, the wetter miombo in the south is relatively well represented in the PA network, but a much smaller percentage (23%) of what is protected is of category PA1.

### Environmental representation

**Fig 3:**
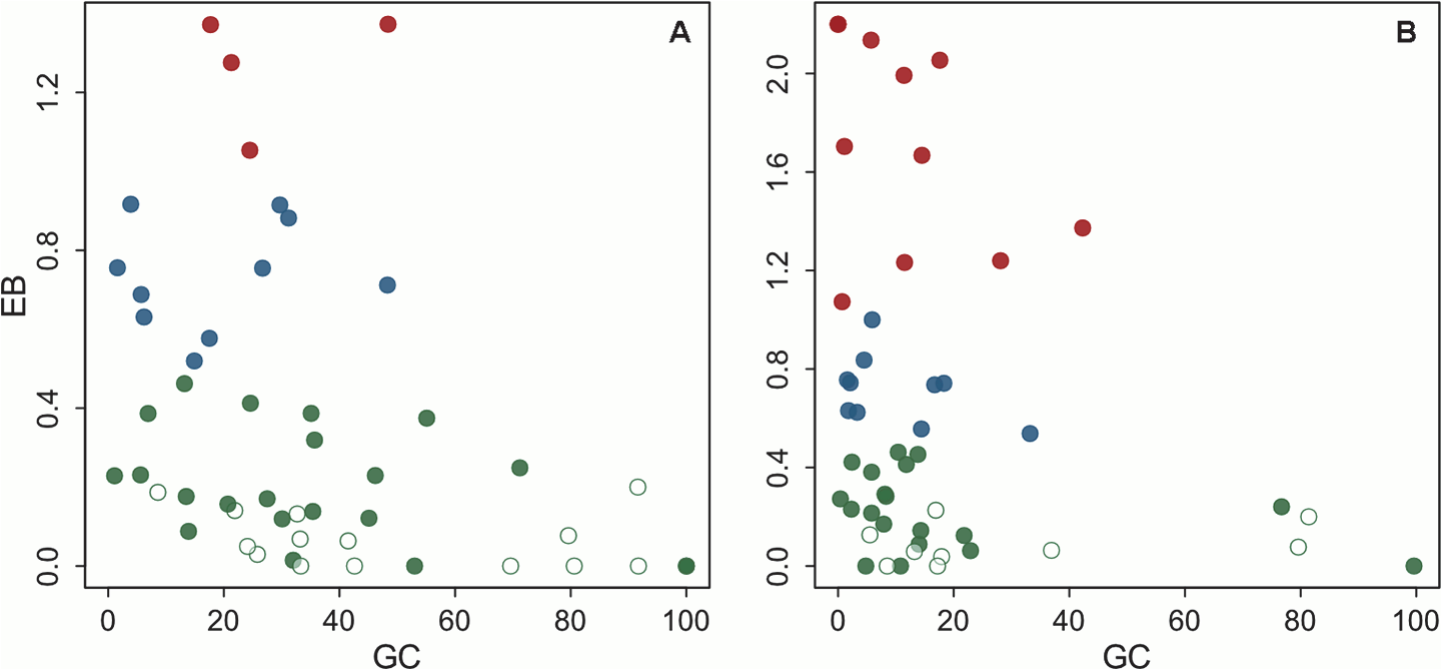
Relationship between geographic coverage and environmental bias in the protected areas network. A) Scatterplot of the percent area protected (GC) and environmental bias (EB) per potential natural vegetation (PNV). The EB was computed as the absolute difference in the median of the MES1 for the protected areas and the whole PNV, divided by the median absolute deviation of MES1 in the PNV (see text for details). The PNVs are grouped in three classes with small (green), intermediate (blue) and large (red) EB values. Open green circles indicate that the EB does not significantly deviate from 0 (Mann–Whitney with Bonferroni adjustment, two-tailed p>0.05). B) As A, but the GC and EB values given for the PA 1 protected areas only.

Large variation in the environmental bias (EB) is observed (Fig 3), indicating that there are clear differences in how representative environmental conditions in the PAs are of those in whole PNVs. This variation is largely independent of the percent area protected of PNVs, except that for the PNVs with a very large percent area protected (>60%) the EB is smaller and less varied, as would be expected. These include all the highland vegetation types and the *Zambezian Kalahari woodlands within edaphic grassland on drainage-impeded or seasonally flooded soils* (Wk/g). The four PNVs where the distribution of the PAs is most biased (EB > 1) are the *Zambezian chipya woodland* (Wy), the *edaphic wooded grassland on drainage-impeded or seasonally flooded soils* (wd), the *Somalia-Masai Acacia-Commiphora deciduous bushland and thicket* (Bd), and the *Climatic grasslands* (G).

PNVs with a geographic coverage by PAs of more than 26% as well as relative high EB values are the *Zambezian chipya woodland* (Wy), *Climatic grasslands* (G), the *edaphic grassland on volcanic soils* (gv), the *Zambezian dry evergreen forest* (Fm), and the *edaphic grassland on drainage-impeded*, *seasonally flooded soils or freshwater swamp* (g/X) (S1 Table). Based solely on their geographic coverage of the PNV alone, these PNVs would be considered low priority for the further assignment of PAs. Yet, the large environmental bias observed means closer examination of conservation efforts is warranted for them.

For many PNVs (23), the environmental bias is larger when only PA1 are considered, with the opposite being true in only eight cases. Both the smaller numbers and the on average larger size of the PA1 areas may partly explain this observation. For most PNVs it is clear that the less strictly PA2 areas complement the nominally stricter PA1 areas by covering environmental conditions not found in the latter.

**Fig 4:**
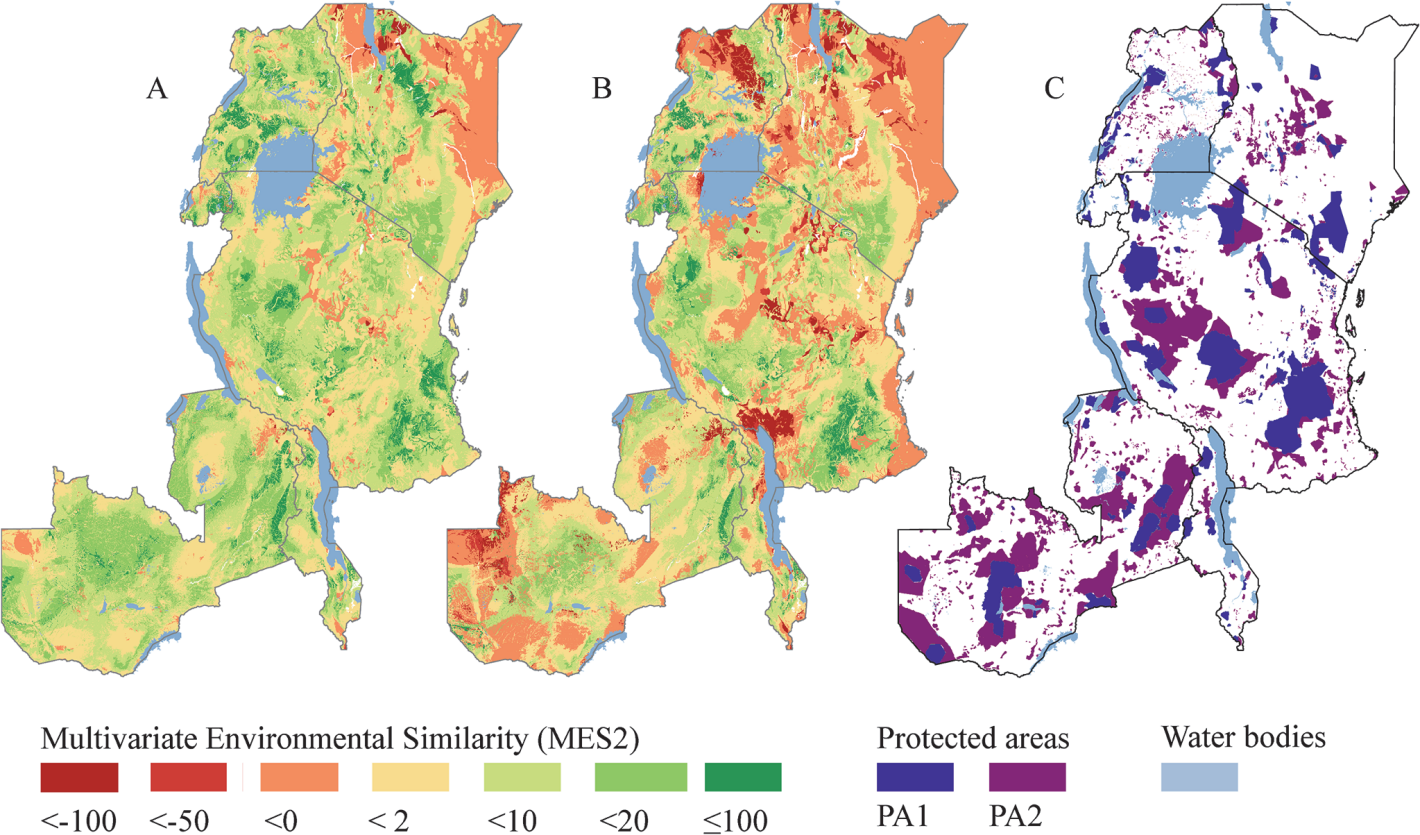
Map of the multivariate environmental similarity (MES2) of the protected areas. A) It combines maps of the 50 PNVs showing how similar environmental conditions in each raster cell are to those in the PA1 + PA2 areas. B) As A, but for PA1 areas only. C) Locations of PA1 and PA2 areas.

The multivariate environmental similarity (MES2) map in Fig 4 shows for each raster cell how similar its environmental conditions are to those in the PAs of the PNV in which the cell is located. It thus identifies areas with environmental conditions that are relatively well represented (green), poorly represented (yellow), or not represented at all (orange-red) in the PA network. For expansion of the network, the orange-red areas will thus best complement existing PAs in terms of their coverage of the environmental conditions in the respective PNVs.

### Human influence

**Fig 5:**
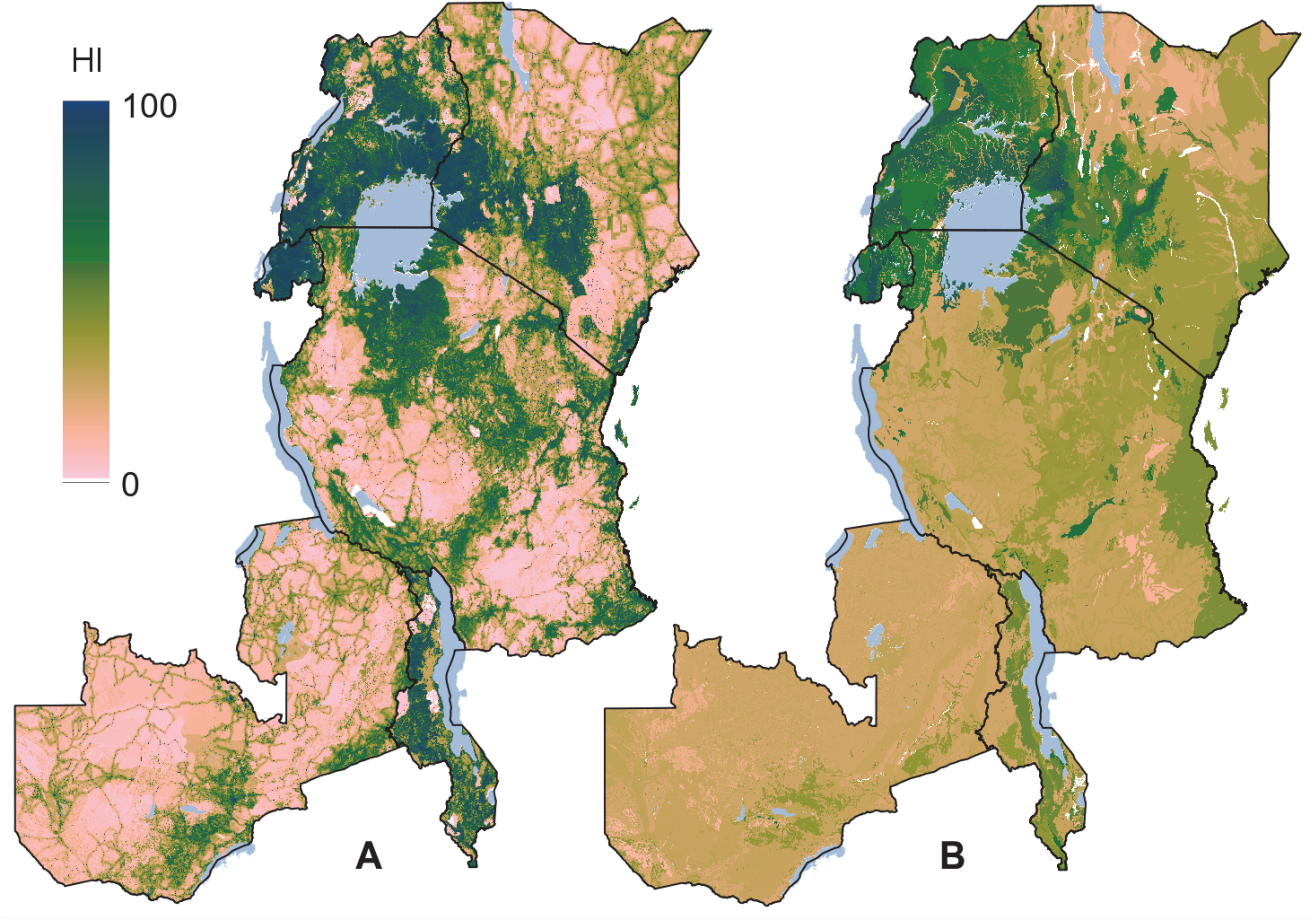
A map of the human influence in eastern Africa. A) Map of the distribution of the human influence index (HI), and B) map of the average human influence (HI_pnv_) by potential natural vegetation type (PNV).

The average human influence (HI_pnv_) (Fig 5) is generally highest for forest PNVs (average = 58 ± 30 standard deviation). This is followed by the bushland, thickets and wooded grassland PNVs (36 ± 26), the open forest and woodlands PNVs (30 ± 26) and the highland vegetation and grasslands PNVs (28 ± 28 and 28 ± 24). The lowest influence is for arid zone PNVs (17 ± 15). Within these PNV groups, there are large differences between PNVs. Those PNVs with the highest HI_pnv_ are located around Lake Victoria and in the highlands of Kenya and northern Tanzania (Fig 5B). These include forests PNVs, such as the *Lake Victoria transitional rain forest* (Ff), *Afromontane moist transitional forest* (Fe), *Lake Victoria drier peripheral semi-evergreen Guineo-Congolian rain forest* (Fi), and the *Afromontane rain forest* (Fa). The *Zambezian dry evergreen forest* (Fm) and the *Single-dominant Widdringtonia whytei forest* (mapped as part of a mosaic with the Montane Ericaceous belt; F/Fc) are the only two forest types that are among the 10 PNVs with the lowest HI_pnv_ scores (S1 Table). Other PNVs with high HI_pnv_ values include the *moist Combretum wooded grassland* (Wcm), Vitellaria wooded grassland (Wb), *Dry Combretum wooded grassland* (Wcd), and *Evergreen and semi-evergreen bushland and thicket* (Be). Human influence is noticeable low in the different miombo woodlands of southern Tanzania and Zambia (Wcd, Wcm; Fig 5B and S1 Table).

There is a broad resemblance between the geographic patterns of human influence and the distribution of PNVs (Fig 5). This is not surprising, as the environmental drivers of agriculture, for example, are also likely to be major determinants of the vegetation distribution. Within PNVs, however, the levels of human influence can also differ considerably.

### Conservation risk

**Fig 6:**
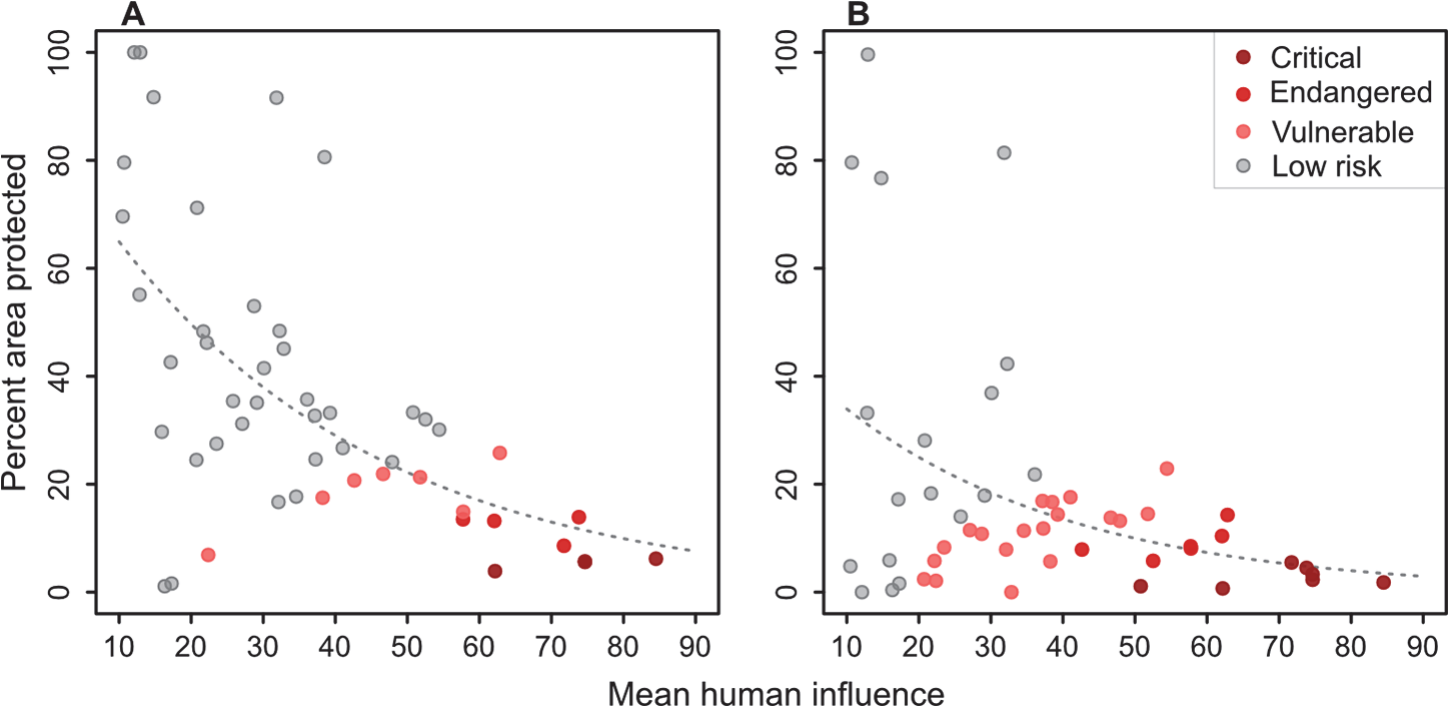
Conservation risk for potential natural vegetations. A) Scatterplot of the average human influence (HI_pnv_) and the percent area protected (GC) for PNVs. We defined the CRI (conservation risk index) as the ratio between the HI_pnv_ and the GC. PNVs with a HI_pvn_ > 50 and a CRI > 10 were classified as critically endangered; PNVs with a HI_pnv_ > 40 and CRI > 4 as endangered and PVNs with a HI_pnv_ > 20 and CRI > 2 as vulnerable. All other PNVs were classified as low risk. Regression statistics: R^2^ = 0.35, p < 0.01. B) As A, but with the GC for the PA1 protected areas only. Regression statistics: R^2^ = 0.14, p = 0.02.

There is a significant negative, although modest relationship between the average HI and the percent area protected (Fig 6A). This suggests a general tendency for protection efforts to be lower in areas of high human influence. For those PAs under strict protection (PA1) only, this relationship is much weaker (Fig 6B).

Nine PNVs stand out for their high HI_pnv_ in combination with very low levels of coverage in the PA network (considering all PAs, of categories PA1 and PA2). Among these, the HI_pnv_ ranges from 58 to 85%, and the area protected ranges from 4 to 14%, indicating that they are at high risks of losing (or have already lost) significant natural vegetation cover. Four of the nine were classified as critically endangered; the *Lake Victoria transitional rain forest* (Ff), the *Afromontane moist transitional forest* (Fe), the *Moist Combretum wooded grassland* (Wcm) and the Vitellaria wooded grassland (Wb). Another four were classified as endangered: the *Afromontane dry transitional forest* (Fh), the *Lake Victoria drier peripheral semi-evergreen Guineo-Congolian rain forest* (Fi), the *Evergreen and semi-evergreen bushland and thicket* (Be) and the *Palm wooded grassland* (P). With a CRI of 3.9, the final PNV of the nine, *Dry Combretum wooded grassland* (Wcd), was classified as vulnerable (Fig 6 and Table 2).

**Table 2:** Classification of potential natural vegetations into three categories of conservation risk according to the criteria presented in the current paper (A) and according to the criteria of Hoekstra et al. [55] (B).

	****Conservation priority****			
	****A****		****B****	
****PNV****	****C1****	****C2****	****H1****	****H2****
Fa	VU	EN		VU
Fb		EN		VU
Fd				
Fe	CR	CR	VU	VU
Ff	CR	CR	VU	CR
Fg		VU		
Fh	EN	CR	VU	VU
Fi	EN	CR	VU	VU
Fm				
Fn		VU		
Fo	VU	VU		
CM	VU	EN		
M		VU		
Wcd	VU	EN	VU	VU
Wcm	CR	CR	VU	EN
Wk		VU		
Wmd				
Wmr		VU		
Wmw		VU		
Wn		VU		
Wo				
Wv				
Wy		VU		
Wmd/Wn				
Bd		VU		
Bds	VU	VU		
Bdw		VU		
Bdw/Wc/g		VU		
Be	EN	EN	VU	VU
bi		VU		
L		CR		VU
P	EN	EN	VU	VU
T/g				
Wb	CR	CR	VU	VU
wd	VU	VU		
We		VU		
Wk/g				
Wmd/Bd				
Wn/g				
A				
Ad				
B		VU		
E				
E/Fc				
D				
S				
G				
g/X		VU		
gm/F	VU	VU		
gv				

C1 and H1 are based on all protected areas; C2 and H2 are based on the PA1 protected areas only.

A) Critically endangered (CR) = PNVs with a conservation risk index (CRI) > 10 and human influence (HI) > 50; Endangered (EN) = PNVs with a CRI > 4 and HI > 40; Vulnerable (VU) = PNVs with a CRI > 2 and HI > 20. B) As A, but with CRI threshold values of 25, 10 and 2, respectively. Potential natural vegetation (PNV) codes are provided in Table 1.

**Fig 7:**
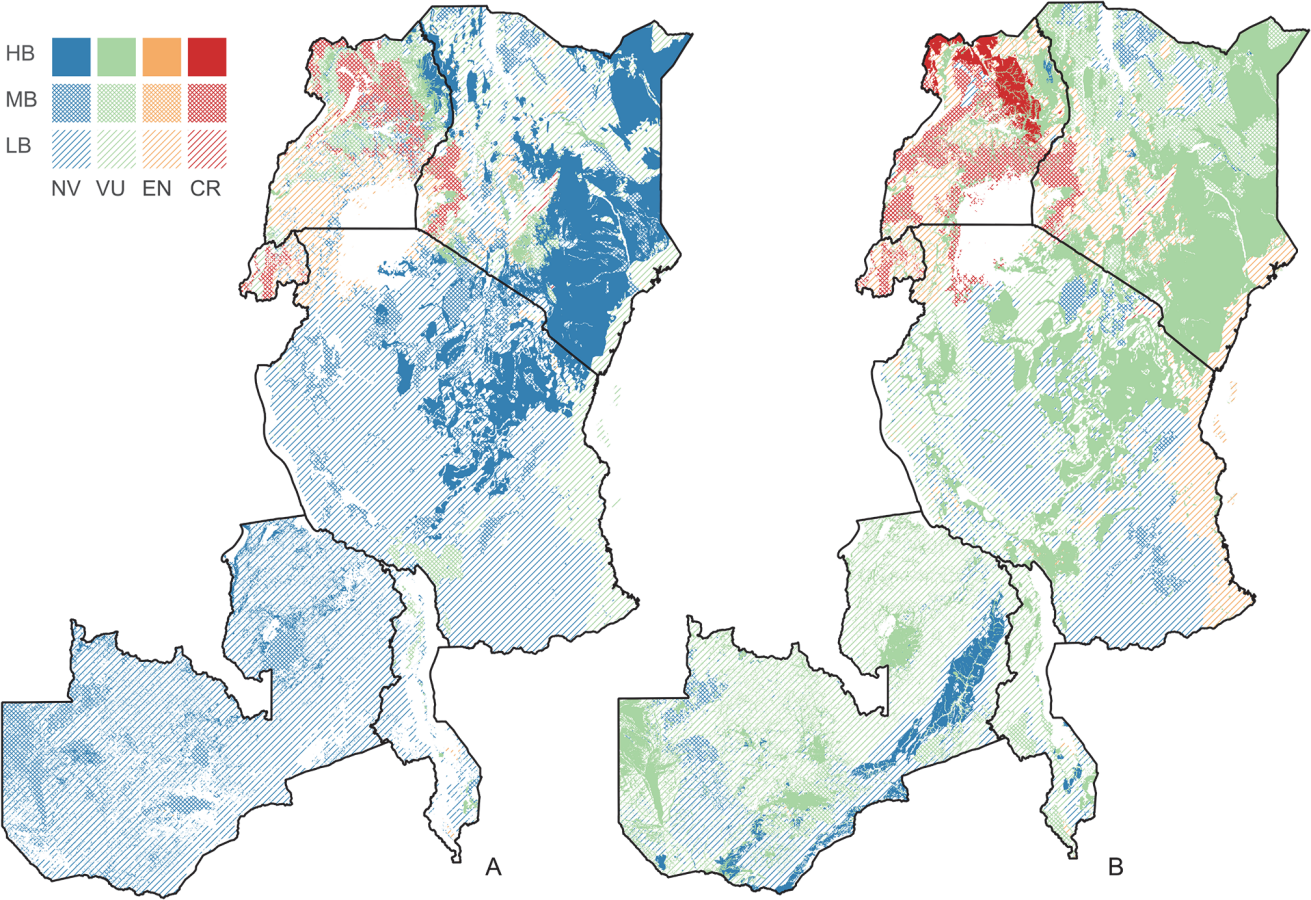
Crisis potential natural vegetations. A) The distribution of the potential natural vegetations (PNVs) classified as critically endangered (CR), endangered (EN), vulnerable (VU), and not vulnerable (NV) and the level of environmental bias (EB). High bias (HB), EB > 1; medium bias (MB), 0.5 ≤ EB < 1; slight or no bias (LB), EB < 0.5. B) As A, but for PA1 areas only.

Although all nine of these PNVs are poorly protected, there are distinct differences in how well PAs represent the variability in environmental conditions within them. For example, the EB of the PAs in the Afromontane dry transitional forest (Fh) and the Evergreen and semi-evergreen bushland and thicket (Be) is 0 and 0.18, respectively, suggesting that the environmental conditions in these PNVs are well represented. On the other hand, the Moist Combretum wooded grassland (Wcm) and the Vitellaria wooded grassland (Wb) PNVs have an EB of 0.69 and 0.9, respectively, a medium level of bias in the distribution of the PAs (S1 Table). These differences may be used to further focus conservation efforts and identify particular problem areas (Fig 7).

The assumption that all IUCN categories are equally effective in conservation in the above calculations may have biased our results. When we repeated the analysis only for strictly protected areas (PA1), the number of PNVs that fell within threat categories CE, EN and VU, increased to 32, with a big increase in particular in the VU category (Table 2 and Fig 6B). In the second analysis, PNVs that shifted from EN to the higher category CR were the *Afromontane dry transitional forest* (Fh) and the *Lake Victoria drier peripheral semi-evergreen Guineo-Congolian rain forest* (Fi). The *Coastal mosaic* (CM), the *Afromontane rain forest* (Fa), the *Afromontane undifferentiated forest* (Fb) and the *Dry Combretum wooded grassland* (Wcd) shifted from VU to EN (Fig 7).

### Regional versus global conservation priorities

**Fig 8:**
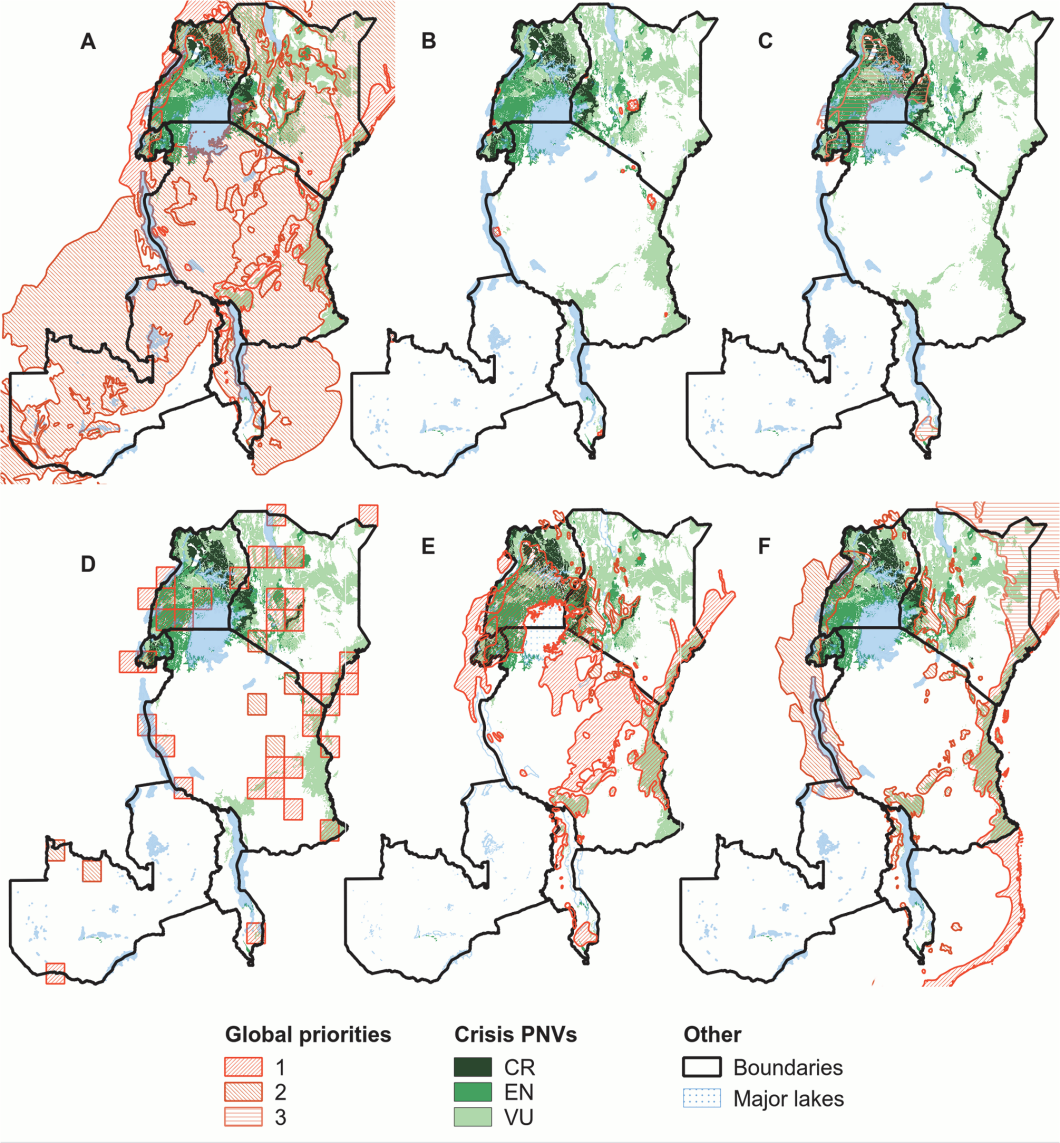
Crisis potential natural vegetations overlaid with global priority areas for conservation. The crisis potential natural vegetations (PNVs) are categorized as critically endangered (CR), endangered (EN) and vulnerable (VU). The map is overlaid with the: A) WWF’s global 200 terrestrial ecoregions map [48]; B) the Centres of Plant Diversity map [89]; C) the Crisis Ecoregions map [55]; D) the conservation priorities for Sub-Saharan Africa map [90]; E) the priorities for conservation intervention in Africa map [41]; and F) the "Biodiversity Hotspots", Conservation International 2011 map [29]. Where relevant, level of priority (1 = highest) is indicated by hatching pattern.

Fig 8 provides a comparison of the distribution of the most endangered PNVs according to our analysis with priority areas for conservation based on four global templates. The WWF’s Global 200 Ecoregions map (G200) [48] covers most of the eastern Africa region. Even so, of our four critically endangered PNVs, it only unambiguously covers the Butyrospermum wooded grassland (Wb). The Centres of Plant Diversity (CPD) map [89] relatively often locates these centres in well protected areas or in areas with low HI. The biodiversity hotspots (BH) map [23,29] overlaps with a number of the vulnerable PNVs, such as the *coastal mosaic* (CM) (which largely overlaps with the Coastal Forests of Eastern Africa hotspot) and the *afromontane rain forest (Fa)* and the *Afromontane forest—grasslands mosaic* (gm/F) (both of which fall within the Eastern Afromontane hotspot). However, there is no overlap with any of the critically endangered PNVs. The Conservation priority areas for Sub-Saharan Africa proposed by da Fonseca et al. [90] overlaps in a few locations with the critically endangered PNVs, but the same 1 degree cells overlap with several other distinct PNVs.

There are also clear differences in areas identified as most endangered by our study and by Hoekstra et a. [55], even though both studies followed a similar approach. The main ecoregion identified as vulnerable by Hoekstra et al. [55] is the Victoria Basin forest-savanna mosaic ecoregion, which only partly overlaps with the critically endangered or endangered PNVs identified in our study (Fig 8C). The differences can be explained by the higher resolution vegetation map used in our study and the fact that on the WWF ecoregion map various PNVs are aggregated into one ecoregion. Another reason is that we used region specific threshold values to classify the PNVs into categories of conservation risk (Table 2).

## Discussion

Conservation efforts in eastern African are remarkable in terms of the percentage of land protected, with the current value of 26% well above the global average of 12.7% [4] and already surpassing the global CBD Aichi target for 2020 of 17%. Conservation efforts, however, cannot be evaluated simply in terms of percentage coverage, but should consider how well different vegetation types are represented [16,91]. Here, we overlaid a high-resolution map of PNVs in eastern Africa onto maps of protected areas, environmental variables, and human influence factors and globally-recognized conservation priorities. We show that there are substantial differences in the conservation status of PNVs. Differences are not only large in terms of the percent area protected, but also in terms of how well the protected areas reflect the environmental variation within vegetation types. These idiosyncratic patterns imply that effective conservation planning and actions require detailed spatial analyses to identify both problems and opportunities in a complex regional and local socio-ecological context.

### Patterns of representativeness in eastern Africa

Our analysis shows that there are large differences in how well eastern African PNVs are represented in the current PA network. Best represented are the Highlands PNVs while the arid zone PNVs are most poorly represented. Both these groups of PNVs have marginal value to agriculture and are remote from population centres. The former have a high distinctiveness value because of their recent evolution and distinctiveness, while the latter (corresponding to the Masai xeric grasslands and shrublands ecoregion on the WWFs ecoregional map [45]) rank low in terms of endemism, richness and non-species biological importance [41]. Poorly represented PNVs can, however, also be found in locations with high biodiversity value, such as the forests, woodlands and bushlands PNVs around Lake Victoria, and in central and northern Uganda, Rwanda, and southwest Kenya. The poor representation of these PNVs can partly be attributed to high human influence (see below) and the subsequent high costs that there would be to forgo other land use options. When taking into account differences in population density and HI_pnv_, however, there are still clear discrepancies in how well particular PNVs are protected in different nations. For an effective translation of regional priorities to national implementation strategies, we need therefore to identify and address country specific factors, be they political, cultural or historical, which influence conservation assessments and prioritization.

The percent area protected is a quick and convenient measure of how well a PNV is represented within the PA network. Most PNVs, however, cover large areas and species and biodiversity patterns will rarely be uniform across them. In consequence, within-PNV variation can influence estimates of how well species or biodiversity patterns are represented in the PA network. Information on biodiversity patterns within the PNVs is not available. Although the level of congruence between environmental diversity (ED) and biodiversity is a subject of debate [92–95], the former measure does provide a proxy for the latter and allows us to infer gaps in the distribution of biodiversity [39,96]. Our result indicate clear differences in how well PAs represent the range of environmental conditions within PNVs. Thus, where the distribution of PAs is biases, geographical coverage alone as a metric may provide an overly optimistic view of the conservation status of PNVs. The situation could be even more serious than we suggest, because edaphic conditions, that potentially vary over much smaller scales, are not accounted for in our analyses.

A combination of maps of the similarity of any given location to the environmental conditions in the PA network and maps showing land availability and human pressure (cf., Fig 4 and Fig **5** in our analysis, respectively), can provide a broad brush overview of available areas whose selection for incorporation into the PA network would increase its representativeness, while minimizing potential land use and rights conflicts. These proposals could subsequently be weighed against a series of other criteria, such as the minimum required size to guarantee the long term persistence of target species or communities [97,98]. Some important future steps to advance this approach are to weigh the pros and cons of the various techniques and methods to measure and express environmental representativeness [94], and to evaluate the extent that vegetation patterns and environmental heterogeneity are congruent with biodiversity patterns, and at what scale [92–96,99]. Promising in that regard are current advances in remote sensing for vegetation, species and biodiversity mapping [100–102].

### Potential natural vegetations at risk

There are considerable differences in the human influence (HI) both between and within PNVs. The PNVs with the highest HI_pnv_ scores are also amongst those that are the most poorly represented in the PA network. There are, therefore, a number of PNVs whose biodiversity and ecological functions are at considerable risk. The four PNVs that stand out in this regard are the critically endangered *Lake Victoria transitional rain forest* [Ff], *Moist Combretum wooded grassland* [Wcm], *Afromontane moist transitional forest* [Fe], and Vitellaria wooded grassland [Wb]). These PNVs are characterized by very high human population densities, a dense road network and an agricultural landscape with relatively few small patches of natural vegetation. Options to expand the current PA network will be limited in most of these PNVs, due to high land use competition. The biotic and abiotic conditions within these PNVs and outside the PAs may also have changed to such a degree that the original vegetation is not likely to recover without significant vegetation restoration measures. Given such challenges, the viable options may be two-fold. The first is to strengthen the management of the most important current PAs, which are defined as priorities by initiatives such as our current study. A potentially controversial approach would be to reduce investments in protected areas in well represented PNVs in favour of increased resources for those in poorly represented PNVs [103]. Ideally information on the effectiveness of activities that support conservation in individual PAs should also be included as a factor in decision-making, as clearly PAs vary enormously in their effectiveness for conservation, with important regional and contextual differences [12,104–108]. Such data is, however, only available for 30% at most of the PAs in eastern Africa [109], and gathering further information is therefore of great practical importance [104,108,110].

Management in PA1 areas may not always be better or more effective in protecting potential natural vegetation [111] and the IUCN classification is not necessarily an indicator of management effectiveness or quality [112]. In our analysis only considering the nominally more strictly protected PA1 areas (an approach used by e.g., Burgess et al. [41]) results in a considerable increase in the number of PNVs that would be classified as vulnerable, endangered, or critically endangered. The percentage of PAs falling into the PA1 category also differed considerably between PNVs. In most PNVs, it should also be noted that the distribution of PA1 areas is considerably more biased than for all PAs together.

The second viable options is to seek solutions that integrate conservation inside and outside PA boundaries [113,114] and that integrate different stakeholders in management [115,116]. This option includes the optimization of matrix management to reduce the effect on populations of target taxa in the conservation units [117–119] through e.g., through the improvement of the connectivity between protected areas and other remaining fragments of natural vegetation and conservation of biodiversity in agricultural landscapes [120].

### Regional versus global priorities

Significant resources for conservation come from global funding mechanisms and it is therefore important to relate how regional and national conservation actions fit within global priorities [10,121,122]. This is illustrated by two global studies [41,55] that used vulnerability as a key criterion to determine that the Victoria Basin forest-savanna mosaic ecoregion is a global priority for conservation. The distribution of this ecoregion corresponds largely with that of the *Lake Victoria transitional rain forest* (Ff) and *Moist Combretum wooded grassland* (Wcm) PNVs, and thus support urgent conservation action. There is an important caveat, however, since this ecoregion also overlaps with a number of other PNVs that are ‘merely’ vulnerable or are not threatened according to our analysis. It is important, then, to focus on the right geographic areas within the ecoregion, which are not necessarily the ‘easiest’ areas for action.

That differences between conservation templates and biodiversity indicators lead to divergent priorities is well documented [122,123]. Our results highlight that scale and resolution of the data are important consideration, with more detailed maps providing significant greater information for planning purposes. There are, however, some limitations to planning based on regional PNV maps such as ours for eastern Africa. One is that some of the vegetation types that occur in the region can also extend far beyond its boundaries of our region (in our case, e.g., the East Sudanian savanna, of which the Wb is part). Without taking this into account the conservation status of these vegetation types may be wrongly approximated. In additions, mismatches in vegetation classifications between the global and regional maps sometimes occur, indicating gaps in our knowledge of local distribution patterns of vegetation and associated species, and these need to be addressed urgently.

In this study we have focussed on threats at the level of the vegetation type, Clearly, other important criteria that need to be considered include: levels of biodiversity [23,29]; endemism and centres of plant diversity [89]; and “irreplaceability” [90]. Estimates are required of how these variables differ across and within PNVs. In earlier studies this has for example been done for the WWF ecoregion classification scheme [41,124]. These estimates, however, were based on species accumulation curves, and not on georeferenced species distribution data. For the PNV classification employed in the current study, information on the total and endemic species numbers remains to be compiles. Such information could, however, be used to adjust national priorities, for example, by attributing higher priorities to PNVs with higher levels of biodiversity or endemism. Clearly, planning must also consider the provision of other ecosystem services, the needs of agriculture and of other livelihood strategies [116]. Poorly planned PA systems that ignore competing interests are likely to lead to conflicts over land and resources [125] and have already led to downgrading, downsizing or even degazetting of large areas in eastern Africa [126]. The way that the benefits and costs of PAs are allocated is crucial [127–129], and the effects of Pa designation on conservation outside boundaries must be understood, since these may be detrimental [130]. Regardless, comparing scenarios for different subsets of PNVs is an important element of the priority-setting process.

## Supporting Information

S1 Appendix Environmental representation. A description of the multivariate similarity index and the environmental bias measure.
(DOCX)

S2 Appendix Human influence. Methods used to compute the human influence
(DOCX)

S1 Fig The potential natural vegetation map of eastern Africa. Potential natural vegetation (PNV) map based on the VECEA PNV map by van Breugel et al. [62]. The full names of the potential natural vegetation types, corresponding to the codes in the legend, are provided in Table 1. PNVs marked with an asterisk were not used in our analysis. For reference purposes the position of capital cities are indicated, with their extent based on the MODIS 2009 urban areas mask [75].
(TIF)

S2 Fig Percent area protected by country. Stacked barplot with with the percent area protected by country within the PA1 (IUCN categories Ib, II, III and IV) and PA2 (IUCN category VI and unclassified) protected areas. The dots and error bars give the average and standard deviation of the human influence by country.
(TIF)

S1 Table Zonal statistics of human influence, geographic coverage and environmental representativeness by PNV. A) Average and standard deviation of the composite human influence (HI) and the individual HI factors, including the accessibility index (AI), the livestock pressure index (LPI), the human population density index (HPI), the vegetation transformation index (VTI), and the percent area converted to croplands (crops). B) Zonal statistics of the geographic coverage (GC) of the potential natural vegetations (PNVs) in the whole region and by country, (EC) the percent area with environmental conditions that are within the range of conditions found in the protected areas, i.e., MES2>0, and (EB) the environmental bias (see the main body of the text for a definition). Statistics were computed for all protected areas (All) and for the PA1 protected areas only. C) The environmental variables used to compute the environmental representativeness (MES2) and EB.
(XLS)

## References

[1] Butchart SHM, Walpole M, Collen B, van Strien A, Scharlemann JPW, Almond REA, et al Global Biodiversity: Indicators of Recent Declines. Science. 2010;328: 1164–1168. 10.1126/science.1187512 20430971

[2] Schipper J, Chanson JS, Chiozza F, Cox NA, Hoffmann M, Katariya V, et al The Status of the World’s Land and Marine Mammals: Diversity, Threat, and Knowledge. Science. 2008;322: 225–230. 10.1126/science.1165115 18845749

[3] Dirzo R, Raven PH. Global State of Biodiversity and Loss. Annu Rev Environ Resour. 2003;28: 137–167.

[4] IUCN, UNEP. World Database on Protected Areas (WDPA) [Internet]. Cambridge, UK: UNEP-WCMC; 2013 Available: http://www.wdpa.org/

[5] Bertzky B, Corrigan C, Kemsey J, Ravilious C, Besançon C, Burgess N. Protected Planet Report 2012: Tracking progress towards global targets for protected areas Gland, Switzerland; Cambridge, UK: IUCN; UNEP-WCMC; 2012.

[6] Neumann RP. Ways of Seeing Africa: Colonial Recasting of African Society and Landscape in Serengeti National Park. Cult Geogr. 1995;2: 149–169. 9225555

[7] Neumann RP. Africa’s last wilderness’: reordering space for political and economic control in colonial Tanzania. Afr-Lond-Int Afr Inst-. 2001;71: 641–665.

[8] Joppa LN, Pfaff A. High and Far: Biases in the Location of Protected Areas. PLoS ONE. 2009;4: e8273 10.1371/journal.pone.0008273#pone.0008273-Ando1 20011603 PMC2788247

[9] Pressey RL, Ferrier S, Hager TC, Woods CA, Tully SL, Weinman KM. How well protected are the forests of north-eastern New South Wales?—Analyses of forest environments in relation to formal protection measures, land tenure, and vulnerability to clearing. For Ecol Manag. 1996;85: 311–333.

[10] Forero-Medina G, Joppa L. Representation of Global and National Conservation Priorities by Colombia’s Protected Area Network. PLOS ONE. 2010;5 10.1371/journal.pone.0013210 PMC2953503 20967270

[11] Scott JM, Davis FW, McGhie RG, Wright RG, Groves C, Estes J. Nature reserves: Do they capture the full range of America’s biological diversity? Ecol Appl. 2001;11: 999–1007.

[12] Bruner AG, Gullison RE, Rice RE, da Fonseca GAB. Effectiveness of Parks in Protecting Tropical Biodiversity. Science. 2001;291: 125–128. 11141563 10.1126/science.291.5501.125

[13] Chape S, Harrison J, Spalding M, Lysenko I. Measuring the extent and effectiveness of protected areas as an indicator for meeting global biodiversity targets. Philos Trans R Soc B Biol Sci. 2005;360: 443–455. 15814356 10.1098/rstb.2004.1592 PMC1569446

[14] Chape S, Spalding M, Jenkins M. The world’s protected areas: status, values and prospects in the 21st century Univ of California Pr; 2008.

[15] Watson JEM, Dudley N, Segan DB, Hockings M. The performance and potential of protected areas. Nature. 2014;515: 67–73. 10.1038/nature13947 25373676

[16] Ladle RJ, Whittaker RJ. Conservation Biogeography. 1st ed. Wiley-Blackwell; 2011.

[17] Duelli P, Obrist MK. Biodiversity indicators: the choice of values and measures. Agric Ecosyst Environ. 2003;98: 87–98.

[18] Pressey R, Cowling R. Reserve selection algorithms and the real world. Conserv Biol. 2001;15: 275–277.

[19] Brooks T, Balmford A, Burgess N, Fjeldså J, Hansen LA, Moore J, et al Toward a Blueprint for Conservation in Africa. BioScience. 2001;51: 613.

[20] Redford K, Coppolillo P, Sanderson E, DaFonseca G, Dinerstein E, Groves C, et al Mapping the conservation landscape. Conserv Biol. 2003;17: 647–647.

[21] Wilson K, Carwardine J, Possingham H. Setting Conservation Priorities. Year Ecol Conserv Biol 2009. 2009;1162: 237–264. 10.1111/j.1749-6632.2009.04149.x 19432651

[22] Rodrigues ASL, Akçakaya HR, Andelman SJ, Bakarr Mi, Boitani L, Brooks TM, et al Global Gap Analysis: Priority Regions for Expanding the Global Protected-Area Network. BioScience. 2004;54: 1092–1100.

[23] Mittermeier RA, Turner WR, Larsen FW, Brooks TM, Gascon C. Global Biodiversity Conservation: The Critical Role of Hotspots In: Zachos FE, Habel JC, editors. Biodiversity Hotspots. Springer Berlin Heidelberg; 2011 pp. 3–22.

[24] Küper W, Sommer JH, Lovett JC, Mutke J, Linder HP, Beentje HJ, et al Africa’s hotspots of biodiversity redefined. Ann Mo Bot Gard. 2004;91: 525–535.

[25] Myers N. Biodiversity hotspots rivisited. BioScience. 2003;53: 916–917.

[26] Zachos FE, Habel JC. Biodiversity Hotspots: Distribution and Protection of Conservation Priority Areas. Springer; 2011.

[27] Balletto E, Bonelli S, Borghesio L, Casale A, Brandmayr P, Taglianti A. Hotspots of biodiversity and conservation priorities: A methodological approach. Ital J Zool. 2010;77: 2–13.

[28] Eken G, Bennun L, Brooks TM, Darwall W, Fishpool LDC, Foster M, et al Key biodiversity areas as site conservation targets. Bioscience. 2004;54: 1110–1118.

[29] Myers N, Mittermeier RA, Mittermeier CG, da Fonseca GAB, Kent J. Biodiversity hotspots for conservation priorities. Nature. 2000;403: 853–858. 10706275 10.1038/35002501

[30] Mittermeier RA, Mittermeier CG, Brooks TM, Pilgrim JD, Konstant WR, da Fonseca GAB, et al Wilderness and biodiversity conservation. Proc Natl Acad Sci U S A. 2003;100: 10309–10313. 12930898 10.1073/pnas.1732458100 PMC193557

[31] Watson J, Fuller R, Watson A, Mackey B, Wilson K, Grantham H, et al Wilderness and future conservation priorities in Australia. Divers Distrib. 2009;15: 1028–1036.

[32] Duputié A, Zimmermann NE, Chuine I. Where are the wild things? Why we need better data on species distribution: Why we need better species distribution data. Glob Ecol Biogeogr. 2014; 23: 457–467

[33] Ferrier S. Mapping Spatial Pattern in Biodiversity for Regional Conservation Planning: Where to from Here? Syst Biol. 2002;51: 331–363. 12028736 10.1080/10635150252899806

[34] Heywood VH. Global Biodiversity Assessiment. Cambridge, UK: Cambridge University Press; 1995.

[35] Boakes EH, McGowan PJK, Fuller RA, Chang-qing D, Clark NE, O’Connor K, et al Distorted Views of Biodiversity: Spatial and Temporal Bias in Species Occurrence Data. PLoS Biol. 2010;8: e1000385 10.1371/journal.pbio.1000385 20532234 PMC2879389

[36] Ferrier S, Powell GVN, Richardson KS, Manion G, Overton JM, Allnutt TF, et al Mapping More of Terrestrial Biodiversity for Global Conservation Assessment. BioScience. 2004;54: 1101–1109.

[37] Moore JL, Balmford A, Brooks T, Burgess ND, Hansen LA, Rahbek C, et al Performance of Sub‐Saharan Vertebrates as Indicator Groups for Identifying Priority Areas for Conservation. Conserv Biol. 2003;17: 207–218.

[38] Margules CR, Pressey RL, Williams PH. Representing biodiversity: data and procedures for identifying priority areas for conservation. J Biosci. 2002;27: 309–326. 12177531 10.1007/BF02704962

[39] Panitsa M, Koutsias N, Tsiripidis I, Zotos A, Dimopoulos P. Species-based versus habitat-based evaluation for conservation status assessment of habitat types in the East Aegean islands (Greece). J Nat Conserv. 2011; 10.1016/j.jnc.2011.04.001

[40] Trakhtenbrot A, Kadmon R. Environmental Cluster Analysis as a Tool for Selecting Complementary Networks of Conservation Sites. Ecol Appl. 2005;15: 335–345.

[41] Burgess ND, Hales JD, Ricketts TH, Dinerstein E. Factoring species, non-species values and threats into biodiversity prioritisation across the ecoregions of Africa and its islands. Biol Conserv. 2006;127: 383–401.

[42] Rodríguez JP, RodríGuez-Clark KM, Baillie JEM, Ash N, Benson J, Boucher T, et al Establishing IUCN Red List Criteria for Threatened Ecosystems. Conserv Biol. 2011;25: 21–29. 10.1111/j.1523-1739.2010.01598.x 21054525 PMC3051828

[43] Lombard AT, Cowling RM, Pressey RL, Rebelo AG. Effectiveness of land classes as surrogates for species in conservation planning for the Cape Floristic Region. Biol Conserv. 2003;112: 45–62.

[44] Bunce RGH, Bogers MMB, Evans D, Halada L, Jongman RHG, Mucher CA, et al The significance of habitats as indicators of biodiversity and their links to species. Ecol Indic. 2013;33: 19–25.

[45] Olson DM, Dinerstein E, D WE, Burgess ND, Powell GVN, Underwood EC, et al Terrestrial Ecoregions of the World: A New Map of Life on Earth. BioScience. 2001;51: 933–938.

[46] Hazen HD, Anthamatten PJ. Representation of ecological regions by protected areas at the global scale. Phys Geogr. 2004;25: 499–512.

[47] Anthamatten P, Hazen H. Unnatural Selection: An Analysis of the Ecological Representativeness of Natural World Heritage Sites*. Prof Geogr. 2007;59: 256–268.

[48] Olson DM, Dinerstein E. The Global 200: Priority ecoregions for global conservation. Ann Mo Bot Gard. 2002;89: 199–224.

[49] Powell GVN, Barborak J, Rodriguez SM. Assessing representativeness of protected natural areas in Costa Rica for conserving biodiversity: a preliminary gap analysis. Biol Conserv. 2000;93: 35–41.

[50] Wikramanayake E, Dinerstein E, Loucks C, Olson D, Morrison J, Lamoreux J, et al Ecoregions in ascendance: Reply to Jepson and Whittaker. Conserv Biol. 2002;16: 238–243. 10.1046/j.1523-1739.2002.01403.x 35701977

[51] White F. The vegetation of Africa: a descriptive memoir to accompany the UNESCO/AETFAT/UNSO vegetation map of Africa by F White Natural Resources Research Report XX [Internet]. Paris: U. N. Educational, Scientific and Cultural Organization; 1983 p. 356 Available: http://www.grid.unep.ch/data/download/gnv031.zip

[52] Burgess N, D’Amico Hales J, Underwood E, Dinerstein E. Terrestrial Ecoregions of Africa and Madagascar: A Conservation Assessment Washington D: World Wildlife Fund Ecoregion Assessments, Island Press; 2004.

[53] Van Breugel P, Kindt R, Lillesø J-PB, Graudal L. An alternative simplified version of the VECEA potential natural vegetation map for eastern Africa Figshare 2015; Available: 10.6084/m9.figshare.1306936

[54] Somodi I, Molnár Z, Ewald J. Towards a more transparent use of the potential natural vegetation concept—an answer to Chiarucci et al. J Veg Sci. 2012;23: 590–595.

[55] Hoekstra JM, Boucher TM, Ricketts TH, Roberts C. Confronting a biome crisis: global disparities of habitat loss and protection. Ecol Lett. 2005;8: 23–29.

[56] Peters H, O’Leary BC, Hawkins JP, Roberts CM. Identifying species at extinction risk using global models of anthropogenic impact. Glob Change Biol. 2015; 21: 618–628. 10.1111/gcb.12749 25236755

[57] Belbin L. Environmental representativeness: Regional partitioning and reserve selection. Biol Conserv. 1993;66: 223–230.

[58] Beggs PJ. New Directions: Climatediversity: A new paradigm for climate science. Atmos Environ. 2013;68: 112–113.

[59] Gordon JE, Barron HF, Hansom JD, Thomas MF. Engaging with geodiversity—why it matters. Proc Geol Assoc. 2012;123: 1–6.

[60] The World Bank. The Worldbank Data [Internet]. 2012 [cited 15 Mar 2012]. Available: http://data.worldbank.org

[61] UN Habitat. The state of African cities: A framework for addressing urban challenges in Africa 2008 Nairobi, Kenya: United Nations Human Settlements Programme; 2008.

[62] Van Breugel P, Kindt R, Lillesø J-PB, Bingham M, Demissew S, Dudley C, et al Potential natural vegetation map of eastern africa: interactive vegetation map for ethiopia, kenya, malawi, rwanda, tanzania, uganda and zambia. Version 1.1 [Internet]. Copenhagen, Denmark; Nairobi, Kenya: Forest and Landscape; World Agroforestry Centre; 2012 Available: http://vegetationmap4africa.org

[63] Van Breugel P, Kindt R, Lillesø JB, Bingham M, Demissew S, Dudley C, et al. Potential Natural Vegetation of Eastern Africa (Ethiopia, Kenya, Malawi, Rwanda, Tanzania, Uganda and Zambia). VOLUME 6: An Overview of The Methods and Material Used to Develop The Map. Forest & Landscape Working Papers 68 [Internet]. Copenhagen: Forest & Landscape, University of Copenhagen; 2011 p. 139. Report No.: 68. Available: http://vegetationmap4africa.org

[64] Wild R, McLeod C, Valentine P. Sacred Natural Sites: guidelines for protected area managers World Conservation Union; 2009.

[65] Overton JM, Leathwick JR. Measuring environmental distinctiveness. Sci Conserv. 2001;174: 1–20.

[66] Elith J, Kearney M, Phillips S. The art of modelling range-shifting species. Methods Ecol Evol. 2010;1: 330–342.

[67] Trabucco A, Zomer RJ. Global Aridity Index (Global-Aridity) and Global Potential Evapo-Transpiration (Global-PET) Geospatial Database [Internet]. Published online, available from the CGIAR-CSI GeoPortal: CGIAR Consortium for Spatial Information (CGIAR-CSI); 2009 Available: http://www.csi.cgiar.org

[68] CGIAR-CSI. CGIAR-CSI SRTM 90m DEM Digital Elevation Database, version 4 [Internet]. CGIAR Consortium for Spatial Information (CGIAR-CSI); 2008 Available: http://srtm.csi.cgiar.org/Index.asp

[69] GRASS Development Team. Geographic Resources Analysis Support System (GRASS GIS) Software, version 7.0 [Internet]. USA: Open Source Geospatial Foundation; 2014 Available: http://grass.osgeo.org

[70] De Wit M, Stankiewicz J. Changes in Surface Water Supply Across Africa with Predicted Climate Change. Science. 2006;311: 1917–1921. 16513946 10.1126/science.1119929

[71] UNEP, Nellemann C, Kullerud L, Vistnes I, Forbes BC, Husby E, et al Globio: global methodology for mapping human impacts on the biosphere [Internet]. Nairobi, Kenya: UNEP-DEWA; 2001 Available: http://www.globio.info/publications

[72] Sanderson EW, Jaiteh M, Levy MA, Redford KH, Wannebo AV, Woolmer G. The human footprint and the last of the wild. BioScience. 2002;52: 891–904.

[73] Fritz S, McCallum I, Schill C, Perger C, Grillmayer R, Achard F, et al Geo-Wiki.Org: The Use of Crowdsourcing to Improve Global Land Cover. Remote Sens. 2009;1: 345–354.

[74] Fritz S, McCallum I, Schill C, Perger C, See L, Schepaschenko D, et al Geo-Wiki: An online platform for improving global land cover. Environ Model Softw. 2012;31: 110–123.

[75] Schneider A, Friedl MA, Potere D. A new map of global urban extent from MODIS satellite data. Environ Res Lett. 2009;4: 044003.

[76] ESA. GlobCover 2005–06 (version 2.2) [Internet]. 2.2 ed. ESA GlobCover Project, led by MEDIAS-France; 2007 Available: http://postel.mediasfrance.org

[77] JRC. Global Land Cover 2000 database version 3 [Internet]. 3rd ed. European Commission, Joint Research Centre; 2003 Available: http://bioval.jrc.ec.europa.eu/products/glc2000/glc2000.php

[78] LP DAAC. MODIS Land Cover Type Yearly L3 Global 500 m SIN Grid (MCD12Q1) [Internet]. Sioux Falls: NASA Land Processes Distributed Active Archive Center (LP DAAC), U.S. Geological Survey (USGS) Earth Resources Observation and Science (EROS) Center; 2009. Available: https://lpdaac.usgs.gov/products/modis_products_table/mcd12q1

[79] Friedl MA, McIver DK, Hodges JCF, Zhang XY, Muchoney D, Strahler AH, et al Global land cover mapping from MODIS: algorithms and early results. Remote Sens Environ. 2002;83: 287–302.

[80] Cincotta RP, Wisnewski J, Engelman R. Human population in the biodiversity hotspots. Nature. 2000;404: 990–992. 10801126 10.1038/35010105

[81] Gorenflo LJ. Human Demography and Conservation in the Apache Highlands Ecoregion, US–Mexico Borderlands In: Cincotta RP, Gorenflo LJ, editors. Human Population—Its Influences on Biological Diversity. Springer; 2011 pp. 153–176.

[82] Kruska RL, Reid RS, Thornton PK, Henninger N, Kristjanson PM. Mapping livestock-oriented agricultural production systems for the developing world. Agric Syst. 2003;77: 39–63.

[83] Linard C, Gilbert M, Snow RW, Noor AM, Tatem AJ. Population Distribution, Settlement Patterns and Accessibility across Africa in 2010. PLoS ONE. 2012;7: e31743 10.1371/journal.pone.0031743 22363717 PMC3283664

[84] Nelson A. Travel time to major cities: A global map of accessibilty [Internet]. Ispra Italy: Global Environment Monitoring Unit—Joint Research Centre of the European Commission; 2008 Available: http://bioval.jrc.ec.europa.eu/products/gam/index.htm

[85] Ahmed AGM, Azeze A, Babiker M, Tsegaye D. Post-drought recovery strategies among the pastoral households in the horn ofafrica: a review Organization for Social Science Research in Eastern and Southern Africa (OSSREA); 2003.

[86] Gryseels G. Role of livestock on mixed smallholder farms in the Ethiopian highlands. A case study from the Baso and Worena Wereda near Debre Berhan. Ph.D., Agricultural University, Wageningen, the Netherlands. 1988.

[87] De Leeuw PN, Rey B. Analysis of current trends in the distribution patterns of ruminant livestock in tropical Africa. World Anim Rev. 1995;83: 47–59.

[88] Wint GRW, Robinson TP. Gridded livestock of the world 2007 Rome: Food and Agricultural Organization of the United Nations, Animal Production and Health Division; 2007 pp 131.

[89] UNEP-WCMC. Centres of Plant Diversity. Version 1.0 (digital reproduction of Centres of Plant Diversity, eds Davis S.D., Heywood V.H. & Hamilton A.C., WWF and IUCN, Gland, Switzerland, 1994–7). 2013.

[90] Da Fonseca GAB, Balmford A, Bibby C, Boitani L, Corsi F, Brooks T, et al Following Africa’s lead in setting priorities. Nature. 2000;405: 393–394. 10839514 10.1038/35013249

[91] Woodley S, Bertzky B, Crawhall N, Dudley N, Londoño JM, MacKinnon K, et al. Meeting aichi target 11: what does success look like for protected area systems? 2012; 23–36.

[92] Allouche O, Kalyuzhny M, Moreno-Rueda G, Pizarro M, Kadmon R. Area–heterogeneity tradeoff and the diversity of ecological communities. Proc Natl Acad Sci. 2012;109: 17495–17500. 10.1073/pnas.1208652109 23045670 PMC3491518

[93] Araújo MB, Humphries CJ, Densham PJ, Lampinen R, Hagemeijer WJM, Mitchell-Jones AJ, et al Would Environmental Diversity be a Good Surrogate for Species Diversity? Ecography. 2001;24: 103–110.

[94] Araújo MB, Densham P, Humphries C. Predicting Species Diversity with ED: The Quest for Evidence. Ecography. 2003;26: 380–383.

[95] Faith DP. Environmental Diversity (ED) as Surrogate Information for Species-Level Biodiversity. Ecography. 2003;26: 374–379.

[96] Brooks T, da Fonseca GA., Rodrigues AS. Species, data, and conservation planning. Conserv Biol. 2004;18: 1682–1688.

[97] Ovaskainen O. Long-Term Persistence of Species and the SLOSS Problem. J Theor Biol. 2002;218: 419–433. 12384046

[98] Margules CR, Pressey RL. Systematic conservation planning. Nature. 2000;405: 243–253. 10821285 10.1038/35012251

[99] Ferrier S, Watson G. An evaluation of the effectiveness of environmental surrogates and modelling techniques in predicting the distribution of biological diversity Environment Australia; 1997 Available: http://www.environment.gov.au/archive/biodiversity/publications/technical/surrogates/

[100] Gould W. Remote Sensing of Vegetation, Plant Species Richness, and Regional Biodiversity Hotspots. Ecol Appl. 2000;10: 1861–1870.

[101] Rocchini D, Balkenhol N, Carter GA, Foody GM, Gillespie TW, He KS, et al Remotely sensed spectral heterogeneity as a proxy of species diversity: Recent advances and open challenges. Ecol Inform. 2010;5: 318–329.

[102] Gillespie TW, Foody GM, Rocchini D, Giorgi AP, Saatchi S. Measuring and modelling biodiversity from space. Prog Phys Geogr. 2008;32: 203–221.

[103] Fuller RA, McDonald-Madden E, Wilson KA, Carwardine J, Grantham HS, Watson JEM, et al Replacing underperforming protected areas achieves better conservation outcomes. Nature. 2010;466: 365–367. 10.1038/nature09180 20592729

[104] Green JM, Larrosa C, Burgess ND, Balmford A, Johnston A, Mbilinyi BP, et al Deforestation in an African biodiversity hotspot: Extent, variation and the effectiveness of protected areas. Biol Conserv. 2013;164: 62–72.

[105] Joppa LN, Loarie SR, Pimm SL. On the protection of “protected areas.” Proc Natl Acad Sci. 2008;105: 6673–6678. 10.1073/pnas.0802471105 18451028 PMC2365567

[106] Hayes TM. Parks, People, and Forest Protection: An Institutional Assessment of the Effectiveness of Protected Areas. World Dev. 2006;34: 2064–2075.

[107] Geldmann J, Barnes M, Coad L, Graigie I, Hockings M, Burgess N. Effectiveness of terrestrial protected areas in reducing biodiversity and habitat loss. cee 10–007. Collaboration for environmental evidence; 2013. Report No.: CEE 10–007. Available: http://www.environmentalevidence.org/SR10007.html

[108] Pfeifer M, Burgess ND, Swetnam RD, Platts PJ, Willcock S, Marchant R. Protected Areas: Mixed Success in Conserving East Africa’s Evergreen Forests. PLoS ONE. 2012;7: e39337 10.1371/journal.pone.0039337 22768074 PMC3387152

[109] Leverington F, Costa KL, Courrau J, Pavese H, Nolte C, Marr M, et al Management effectiveness evaluation in protected areas—a global study Second edition 2010. Brisbane, Australia: The University of Queensland; 2010 p. 87 Available: http://www.esee2009.si/papers/Mauerhofer-The_-Convention-Check.pdf

[110] Rodrigues ASL, Andelman SJ, Bakarr MI, Boitani L, Brooks TM, Cowling RM, et al Effectiveness of the global protected area network in representing species diversity. Nature. 2004;428: 640–643. 15071592 10.1038/nature02422

[111] Nelson A, Chomitz KM. Effectiveness of Strict vs. Multiple Use Protected Areas in Reducing Tropical Forest Fires: A Global Analysis Using Matching Methods. PLoS ONE. 2011;6: e22722 10.1371/journal.pone.0022722 21857950 PMC3156699

[112] Leroux SJ, Krawchuk MA, Schmiegelow F, Cumming SG, Lisgo K, Anderson LG, et al Global protected areas and IUCN designations: Do the categories match the conditions? Biol Conserv. 2010;143: 609–616.

[113] DeFries R, Hansen A, Turner BL, Reid R, Liu J. Land use change around protected areas: management to balance human needs and ecological function. Ecol Appl. 2007;17: 1031–1038. 17555216 10.1890/05-1111

[114] Cox RL, Underwood EC. The Importance of Conserving Biodiversity Outside of Protected Areas in Mediterranean Ecosystems. PLoS ONE. 2011;6: e14508 10.1371/journal.pone.0014508 21249126 PMC3017544

[115] Bray DB, Merino-Perez L, Negreros-Castillo P, Segura-Warnholtz G, Torres-Rojo JM, Vester HFM. Mexico’s Community-Managed Forests as a Global Model for Sustainable Landscapes. Conserv Biol. 2003;17: 672–677.

[116] Adams WM, Hulme D. If community conservation is the answer in Africa, what is the question? Oryx. 2001;35: 193–200.

[117] Lindenmayer DB, Wood JT, Cunningham RB, Crane M, Macgregor C, Michael D, et al Experimental evidence of the effects of a changed matrix on conserving biodiversity within patches of native forest in an industrial plantation landscape. Landsc Ecol. 2009;24: 1091–1103.

[118] Tikkanen O-P, Heinonen T, Kouki J, Matero J. Habitat suitability models of saproxylic red-listed boreal forest species in long-term matrix management: Cost-effective measures for multi-species conservation. Biol Conserv. 2007;140: 359–372.

[119] Newmark WD. Isolation of African protected areas. Front Ecol Environ. 2008;6: 321–328.

[120] Scherr SJ, McNeely JA. Biodiversity conservation and agricultural sustainability: towards a new paradigm of “ecoagriculture” landscapes. Philos Trans R Soc B Biol Sci. 2008;363: 477–494. 17652072 10.1098/rstb.2007.2165 PMC2610165

[121] Rodríguez JP, Taber AB, Daszak P, Sukumar R, Valladares-Padua C, Padua S, et al Globalization of conservation: a view from the South. Science. 2007;317: 755 17690278 10.1126/science.1145560

[122] Brooks TM, Mittermeier RA, da Fonseca GAB, Gerlach J, Hoffmann M, Lamoreux JF, et al Global Biodiversity Conservation Priorities. Science. 2006;313: 58–61. 16825561 10.1126/science.1127609

[123] Funk SM, Fa JE. Ecoregion Prioritization Suggests an Armoury Not a Silver Bullet for Conservation Planning. PLoS ONE. 2010;5: e8923 10.1371/journal.pone.0008923 20111722 PMC2811746

[124] Kier G, Barthlott W. Measuring and mapping endemism and species richness: a new methodological approach and its application on the flora of Africa. Biodivers Conserv. 2001;10: 1513–1529.

[125] Robbins P. Political Ecology. Second edition John Wiley & Sons; 2012.

[126] Mascia MB, Pailler S, Krithivasan R, Roshchanka V, Burns D, Mlotha MJ, et al Protected area downgrading, downsizing, and degazettement (PADDD) in Africa, Asia, and Latin America and the Caribbean, 1900–2010. Biol Conserv. 2014;169: 355–361.

[127] Adams WM, Hutton J. People, Parks and Poverty: Political Ecology and Biodiversity Conservation. Conserv Soc. 2007;5: 147.

[128] Brooks JS, Franzen MA, Holmes CM, Grote MN, Mulder MB. Testing Hypotheses for the Success of Different Conservation Strategies. Conserv Biol. 2006;20: 1528–1538. 17002770 10.1111/j.1523-1739.2006.00506.x

[129] Thompson M, Homewood K. Entrepreneurs, elites, and exclusion in Maasailand: Trends in wildlife conservation and pastoralist development. Hum Ecol. 2002;30: 107–138.

[130] Sutherland WJ, Adams WM, Aronson RB, Aveling R, Blackburn TM, Broad S, et al One Hundred Questions of Importance to the Conservation of Global Biological Diversity. Conserv Biol. 2009;23: 557–567. 10.1111/j.1523-1739.2009.01212.x 19438873

