# Supplementary material for: Environmental Gap Analysis to Prioritize Conservation Efforts in Eastern Africa
Source: PLoS One. 2015 Apr 9;10(4):e0121444. doi: 10.1371/journal.pone.0121444 (PMC4391866; doi:10.1371/journal.pone.0121444)
Supplement: S1 Appendix — A description of the multivariate similarity index and the environmental bias measure. (DOCX) [file pone.0121444.s001.docx]

# S2 Appendix. Environmental representation.

The multivariate environmental similarity (MES) calculation represents how similar a point is to a reference set of points (N), with respect to a set of predictor variables (V1, V2, ...). The similarity of a point P to N with respect to variable V is computed as the deviation from the median of V in N. The following is based on the explanation by Elith et al. [1]. Suppose p_i_ is the value of variable V at point P, min_i_ the minimum value of V in N, max_i_ the maximum value of V in N, and V_m_ the median of V in N. The deviation is calculated as:

1. 2 * the percentage of N with V that is smaller than p_i_ if p_i_ ≤ V_m_ and p_i_ > min_i_.
2. 2 * the percentage of N where V is larger than p_i_ if p_i_ > V_m_ and p_i_ < max_i_.
3. (p_i_ – min_i_) / (max_i_ – min_i_) * 100 if p_i_ < min_i_
4. (max_i_ – p_i_) / (max_i_ –min_i_) * 100 if p_i_ > max_i_

The final multivariate similarity for P is computed as the minimum of the similarity values with respect to each of the variables. Amongst others uses MES is employed to evaluate the validity of extrapolating environmental distribution models to new areas or novel climates.

## MES1 and MES2

For our study we created two different MES surfaces for each potential natural vegetation (PNV); MES1 and MES2. MES1 was computed for each raster cell and indicates how similar its environmental conditions are to the overall conditions of the PNV. Thus, for a given PNV, the reference set of points (N) consists of all raster cells within that PNV. For each raster cell (P) in the PNV, the environmental values are compared to those in N, following the calculations above. MES2 was computed for each raster cell and indicates how similar its conditions are to those found in the PAs of the PNV. Thus, for a given PNV, the reference set of points (N) consists of all raster cells within the protected areas in that PNV.

For MES2 we used the original approach as outlined above. For MES1 we were interested in the similarity of the conditions inside and outside the PAs across all environmental gradients. We therefore combined the similarity values of the individual variables using the average rather than the minimum to take into account all variables.

## EB

The Environmental Bias (EB) takes the MES1 surface and compares how the median MES1 for the whole PNV differs from the median MES1 for the PAs. It is computed as the absolute difference of the median of the MES for the whole target area (MES_A_) and the median of the MES for the subset (MES_B_), divided by the median absolute deviation (MAD ) of MES_A_ (Figure 1).


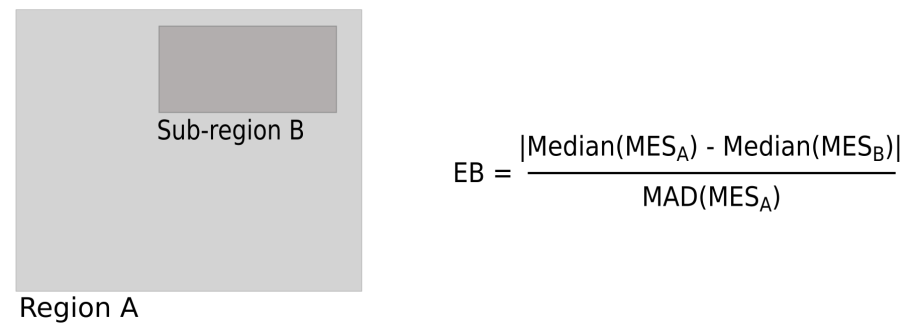


Figure 1. Environmental bias of sub-region B within region A

## Tools

To compute the MES and EB we created the r.mess and r.meb add-ons, respectively, for GRASS GIS 7.0. Both r.mess and r.meb are available in GRASS 7 from the g.extension menu or from <http://grasswiki.osgeo.org/wiki/AddOns/GRASS7/>.

Alternative tools to compute the MES are the Maxent software (<http://www.cs.princeton.edu/~schapire/maxent/>) that originally implemented this statistic and the dismo package for R (<http://cran.r-project.org/web/packages/dismo/>) with the function mess.

## References

1. Elith J, Kearney M, Phillips S. The art of modelling range-shifting species. Methods Ecol Evol. 2010;1:330–342.
