# Supplementary material for: Environmental Gap Analysis to Prioritize Conservation Efforts in Eastern Africa
Source: PLoS One. 2015 Apr 9;10(4):e0121444. doi: 10.1371/journal.pone.0121444 (PMC4391866; doi:10.1371/journal.pone.0121444)
Supplement: S2 Appendix — Methods used to compute the human influence (DOCX) [file pone.0121444.s002.docx]

Appendix S3 Human influence

# A composite map of the human influence

We used a two-step approach to estimate the human influence on the natural vegetation, summarized in Figure 1 in the main text.

**First step - estimate of loss of potential natural vegetation cover**: We estimated the percentage of converted areas of natural vegetation using the global cropland map [1,2] available from <http://beta-hybrid.geo-wiki.org/>), the MODIS 2009 urban areas mask [3] and the VMap0 Roads vector layer (available from <http://gis-lab.info/qa/vmap0-eng.html>). We assigned a conversion score of 100 to areas converted to urban areas or roads. The conversion score for areas converted to croplands was equal to the percentage of land converted according to the crop mask. Note that in this step only the direct effects (removal of vegetation cover) of roads are accounted for. The resulting map is given in Figure 1 (AI).


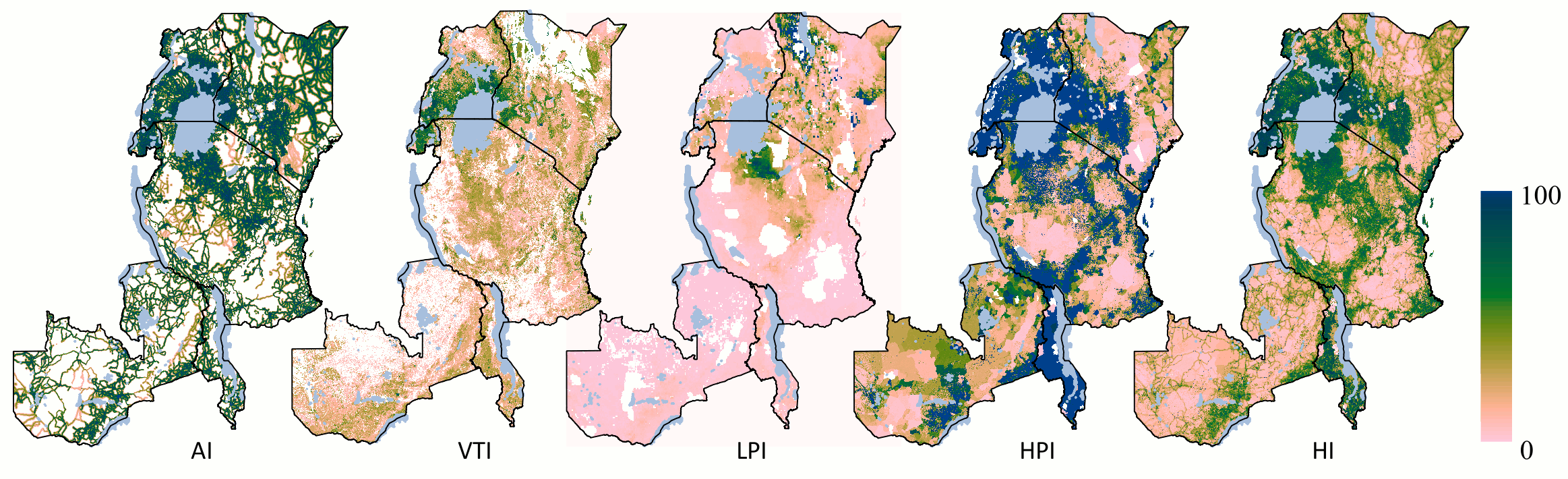


Figure 1. Map of the human influence (HI) and the maps of the individual human influence factors used to create this layer following the steps in Figure 1 in the main text. The AI represents the travel time to the nearest area with a human population densities ≥ 1000 people /km^2^. The VTI is the vegetation cover change index, LPI the livestock pressure index, HPI the human population density and croplands the percentage of land converted to crop lands. All layers have standardized scores from 0 (no influence) to 100 (very high human influence or total removal of the potential natural vegetation cover).

**Second step – estimate of pressure on remaining natural vegetation**: We calculated the relative human influence [4] for areas with natural vegetation (conversion scores lower than 100). As proxy indicators for human influence, we used human population density, travel distance to high population density area, livestock pressure and the relative change in the vegetation physiognomy between potential and actual vegetation cover. Each factor received a score from 0 to 100, representing a scale of no to maximum influence (removal or replacement of the potential natural vegetation cover). For each raster cell, the average of the four human influence scores was multiplied with the percentage of non-converted area. We did not take into account possible interactions of threat. There is no consistent and detailed enough data available to make justifiable assumptions, so we opted to keep the rules used as simple as possible. The maps of the individual HI layers and the final HI layer are given in Figure 1 (VTI, LPI, HPI).

**Third step – combining the output of first two steps**: This human influence score for non-converted areas was added to the conversion score, resulting in the human influence layer (HI) for the whole region used in the main text (Figure 1, HI map).

Our index provides an estimate of the loss of potential natural vegetation cover (first step) and potential degradation due to human pressure of the potential natural vegetation cover (second step). We do not imply that agricultural or urban areas have no biodiversity or other values. The HI score provides an indication of the extent to which the potential vegetation has been replaced by urban areas, croplands or secondary vegetation. However, each of these land cover types represents their own set of biodiversity and ecosystem values.

The factors we used only provide an incomplete description of the human influence on our natural resources. Other potentially important factors, such as pollution, global warming or invasion of pest and exotics were not included because we lacked the data. For this analysis we therefore focused on the direct impact of human land use and infrastructure and the expected influence of human population density on the potential natural vegetation cover. We acknowledge, however, that this may constitute an underestimation of the human impact on the natural vegetation, especially in the long term.





Figure 2. The vegetation cover change (VTI) estimates based on a comparison of four global land cover maps (see text) and the potential natural vegetation map for eastern Africa. The fifth layer shows the average over the four layers.

In the following sections, we describe in more detail how we created the 4 human influence factors provided in Figure 1.

# Changes in the potential natural vegetation cover

As a proxy indicator for the change in the natural vegetation, we compared the physiognomy of the potential natural vegetation distribution with the physiognomy of the current vegetation cover. There are various land cover and use maps available. They all exhibit classification uncertainties, potentially resulting in significant amounts of spatial disagreement across land cover products classifications [5–10]. To reduce the level of uncertainty, we used corroborating evidence of four different land cover maps. These include the GlobCov regional land use cover map version 2.2 [11,12], the Global Land Cover 2000 (GLC2000) for Africa, version 3 [13] and the MODIS Land Cover data, Land Cover Type 1, IGBP global vegetation classification scheme for 2005, and the MODIS Land Cover data, Land Cover Type 1, University of Maryland (UMD) scheme [14,15].

Differences in definitions and classification criteria used in the four LUC maps and the PNV map makes comparison between the actual vegetation mapped on the LUC maps and the potential vegetation a challenge. Especially in the transition zones one can see a high regional variability between LUC maps [9]. In addition, there is a considerable natural variability in physiognomic vegetation cover within the PNVs and the existence of different stable states (see e.g., the Serengeti). Because of this variability and the uncertainty in the data sets, the land cover data and PNV map were aggregated in 7 broad land cover classes based on the description of the original land cover classes. These include two classes of completely converted land; (U) urban and (A) agricultural or forest plantations, and five physiognomic vegetation classes; (FO) forest, (OF) open forest or woodland, (BT) bushland, thicket and wooded grassland, (GH) grasslands and herbaceous cover and (BS) stunted bushland, and semi-desert (Table 1, Table 2, Table 3, Table 4). The PNV classes were reclassified in the same broad physiognomic classes (Table 6). Next, values of 25, 50, 75 and 100 were assigned when the physiognomy of the LUC map was respectively 1, 2, 3 or four steps below the physiognomy of the PNV map, in the sequence FO → OF → BT → GH → BS. If an area was marked as agriculture or urban land, it also was assigned the maximum score of 100. In case of compound mapping units with two or more land cover types, scores were calculated for each land cover type separately and then an average score was calculated weighted according to the percentage coverage of each land cover type. We will henceforward refer to these differences in physiognomy between the potential and actual vegetation cover as the vegetation transformation index (VTI).

It should be noted that there is an inherent bias in the approach described above. It is only suitable to identify changes up to, but not including BS. Moreover, the highest possible score is not the same for all physiognomic types; the maximum score for GH is only 1, and for FO the maximum score is 4. This means that one should be cautious when comparing VTI scores between different physiognomic classes. For more detailed local studies, one would ideally include different measures of vegetation degradation.


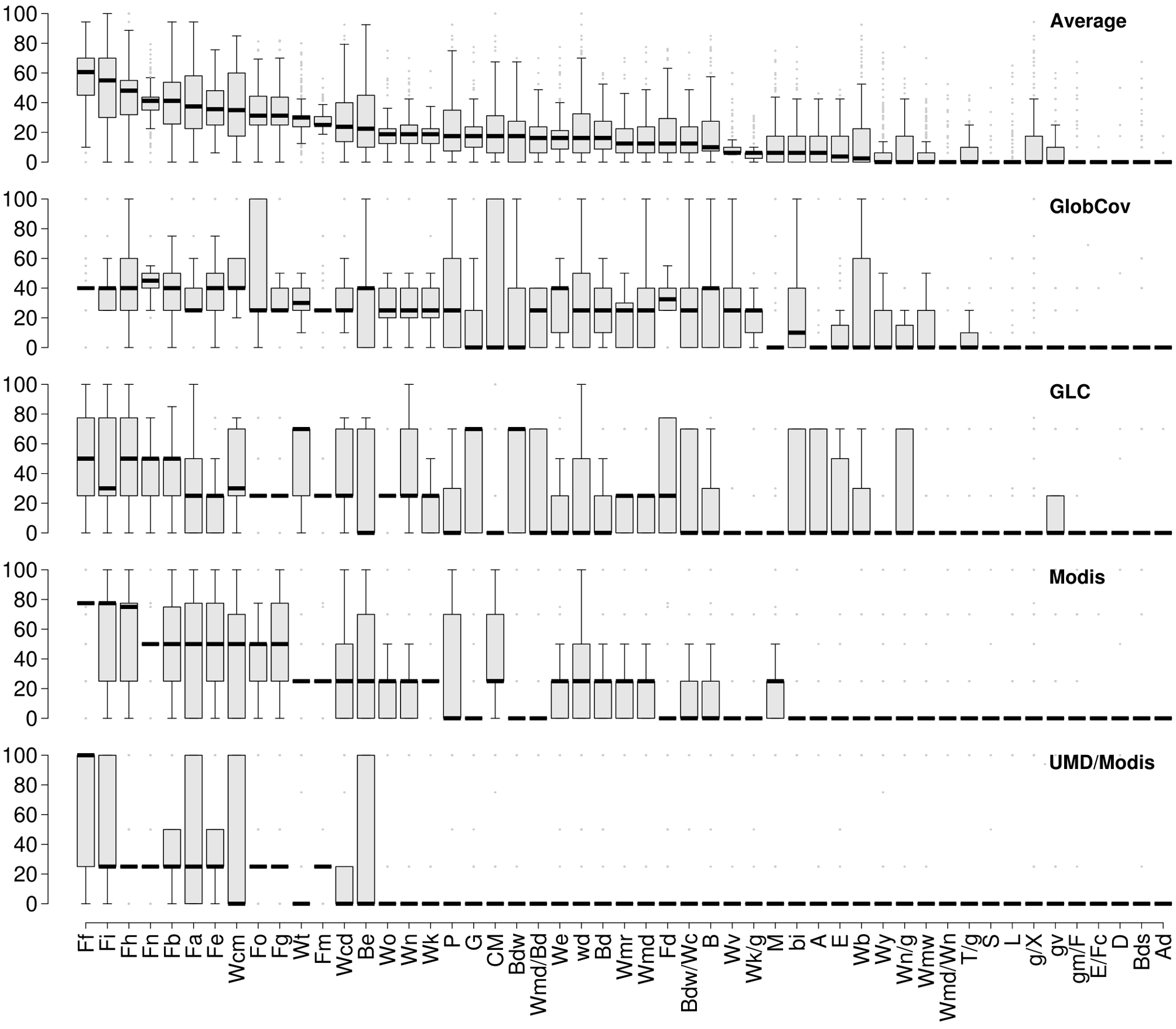
Figure 3. Distribution of VT scores per potential natural vegetation types (PNVs), ranked according to the median VTI scores. VTI scores were computed using, from up to down, the Global cropland map (IIASA), the GlobCov regional land use cover map version 2.2 (GlobCov), the Global Land Cover 2000 for Africa, version 3 (GLC), the MODIS Land Cover data, Land Cover Type 1, IGBP global vegetation classification scheme for 2005 (Modis), and the MODIS Land Cover data, Land Cover Type 1, University of Maryland (UMD) scheme (UMD/Modis).

The individual VTI maps (first four maps in Figure 2) show clear deviating patterns. There are marked differences in e.g., the estimated extent of land transformation in the coastal regions, the Kenyan highlands and Uganda, central Tanzania and Malawi. Differences are sufficiently large to result in different rankings of the PNVS according to their median VTI score (Figure 3). For more local or detailed studies, it may be advisable, especially in areas where the different LUC maps show strong disagreements, to examine the different land use maps in more detail to select the ones most accurate for your target area.

# Travel time to urban centers

Human access can be expressed as a distance measure. However, there are various factors, such as infrastructure and terrain characteristics that impede access to areas. To take these into account measures of travel time rather than the absolute distance are more appropriate [16].

We calculated a travel time from urban centers to any point in the rural or natural areas using the r.cost function in GRASS GIS 7.0 [17]. This function computes the cumulative cost of moving between different geographic locations on an input raster map whose cell category values represent cost. Costs are derived from several spatial data sets (friction layers) that represent roads, terrain, land cover and other geographic features that should be considered when estimating the travel time to the target location. We followed the method outlined by Nelson [18] with some modifications, as explained below.

## Road based travel friction layer (rbts)

As input we used the VMap0 Roads Reference (from <http://gis-lab.info/qa/vmap0-eng.html>). Unlike [18] we did not include rivers and railways. To our best knowledge, rivers are generally not used for transport in this part of the world while railways are few. They only connect the main urban centers, while we are concerned with travel times from urban centers to rural and natural areas outside the urban centers. We converted the road vector layer to a 300m resolution raster layer and assigned average travel speeds depending on the type of road (Table 5).

## Off-road foot based travel (ofbt)

Potential off-road foot based travel speed was assumed to depend on the land cover type. As input we used the GLC 2000 land cover layer [13] and converted the land cover classes to travel speeds (km/h) following the conversion factors in Table 7. The resulting layer *pfbt* was divided by a correction factor to account for the effect of slope on the travel speed [19]. The resulting layer was *ofbt*.

$$ofbt = \frac{pfbt}{e^{-ks}}$$

The factor *k* defines the effect of slope on travel speed. We assumed *k* to be 3 and constant for uphill and downhill following [18]. The slope (*s*) was calculated based on the 3'' SRTM digital elevation model [20] using the r.slope.aspect function of GRASS GIS.

Rivers and other water bodies in general represent barriers, except where there are bridges. Because the time to cross a river depend on e.g., on width and depth, information we do not have, we assumed a fixed value of 60 minutes/km to cross water bodies. We used the AEON river database [21] whereby we reclassified all lines representing perennial rivers as 60 and other lines as 0. The vector layer was subsequently converted to a raster layer (*rfl*) with a resolution of 300 m. Note that when two adjacent cells representing the rivers are connected diagonally they may not function as a barrier at that point as r.cost algorithm allows for diagonal movement. To remedy this, an anti-aliasing filter was applied.

## Combining friction layers

We combined the road based travel layer (*rbts*) and the off-road foot based travel layer (*ofbt*) using the maximum value of the two. Next, we added up the resulting layer and the river friction layer (*rfl*) to get the travel friction surface (*tfs*):

$$tfs=maximum\left( rbts, ofbt \right)+rfl$$

## Cumulative travel cost service

We used the Afripop database [22], selecting all areas with human population densities >= 1,000, and the rasterized VMap0 Populated Place Polygon Reference map with locations of settlements and towns (<http://gis-lab.info/qa/vmap0-eng.html>) to create a new raster layer with urban areas. Next, we calculated for each layer the cumulative travel time (in hours) to the urban centers, using the combined travel friction layers (*tfs*) as input.

## Human influence based on travel time to nearest urban center

How human influences scale with travel distance to the nearest urban centers is largely unknown. For our analysis, we made the assumption that areas that can be realistically reached by people from towns (or vice versa) within six hours are the ones that are likely to be affected most by human pressure and that human pressure increases with decreasing travel times.

A linear increasing score from 0 to 100 was assigned for travel times between 6 and 0 hours, and a score of 0 to all areas further than 6 hours from the nearest urban area. We will refer to this factor as the accessibility index (AI).

# Livestock pressure

As a proxy indicator of the pressure exerted by livestock on the environment [23] we computed the ratio of feed requirement and feed availability. The main livestock species in east Africa in the (semi-)natural areas are cattle, goats and sheep, while especially in the drier regions there are also considerable numbers of camels, donkeys, and horses [24–26]. We used the cattle, goat, and sheep density layers from the FAO [27], which give the estimated livestock densities corrected for unsuitability and adjusted to match FAOSTAT totals for the year 2005. Data for the camels, donkeys and horses was not available, so are estimations are likely to be too conservative.

## Feed requirements

The livestock numbers were converted to tropical livestock units (TLU), with one TLU being equivalent to 250 kg body-weight. In sub-Saharan Africa, one TLU is assumed to be equal to 0.7 cow, 0.1 sheep, and 0.1 goats respectively. Next, feed requirements were estimated to be 5, 10 and 10 kg per TLU per day for maintenance for cattle and sheep and goats respectively [28] and double that when factoring in growth, work, lactation, reproduction, herd structure, and thermoregulation [29]. Thus, the total DMP / km^2^ for feed requirements (FR) were calculated as:

$$FR=365\times({TLU}_{cattle}\times10+({TLU}_{sheep}+{TLU}_{goats})\times20)$$

This estimation ignores diet differences between species [30,31] or differences in feed intake between seasons and vegetation types [28]. However, most of the variance in feed requirements across the region is determined by the livestock numbers [32] so our estimations are sufficient to provide a broad brush overview of patterns in livestock feed requirements across the region.

## Feed availability

We used the decadal dry matter production data (DMP) for the period 1999 - 2011 from the MARS-FOOD project [33] as a proxy for dry matter availability. DMP provides an indication of the dry matter biomass increase (growth rate) and is directly related to the well-known NPP (Net Primary Productivity), but customized for agro-statistics and expressed in kilograms of dry matter (kgDM) per hectare per day. Based on the decadal data, we calculated the average annual dry matter productivity (DMP_yr_).

To maintain ecosystem functioning and avoid undesirable changes in vegetation composition or degradation, a certain portion of the standing biomass should be maintained. Maximum use of available range is commonly set at 25–50% of the available range biomass, the rate of use depending on the fragility of the ecosystem [34–36]. We assumed permissible off-take values (PO) of 25, 35, 40, 45 and 50% of the DMP in the hyper-arid, arid, sub-humid and humid zones, respectively. These zones were defined based on the aridity index (AI) using the following threshold values: hyper-arid if AI < 0.03; arid if 0.03 < AI < 0.20; semi-arid if 0.20 < AI < 0.50; sub-humid IF 0.50 < AI < 0.65 and humid if AI > 0.65. The AI, defined as the ratio between the mean annual precipitation and the mean annual potential evapotranspiration, was obtained from the CGIAR-CSI Global-Aridity and Global-PET Database [37,38].

## Livestock pressure index

The relative livestock grazing and browsing pressure on natural vegetation was calculated as 1 minus the ratio of livestock feed requirements and feed availability, whereby the maximum feed requirement was set to be equal to the DMP_yr_. The equation below summarizes how the relative livestock pressure index (LPI) was calculated:

$$LPI=1-\frac{{DMP}_{yr}\times PO-FR}{{DMP}_{yr}\times PO} if {DMP}_{yr}-FR\geq0$$

$$LPI=1-\frac{{DMP}_{yr}\times PO-{DMP}_{yr}}{{DMP}_{yr}\times PO} if {DMP}_{yr}-FR<0$$

Here, DMP_yr_ is the annual dry matter production, PO the permissible off-take and FR the feed requirements. Under the given assumptions, negative numbers indicates that current feed requirements exceed what can be sustainably provided by the natural vegetation. Note, however, that it does not take into account the use of imported feed or migration of livestock to other areas in times of feed shortages as such information is not available for most parts of the region. On the other hand, using average DMP_yr_ values may result in underestimation of the influence of livestock on the vegetation. The final scores between 1 (high pressure) and 0 (no pressure) were converted to a scale from 0 to 100, while values below 0 were held constant at 100.

# Human population

The number of people in a given area is frequently cited as a primary cause of declines in species and ecosystems [39], with higher human densities leading to higher levels of influence on nature. However, how human influences scale with human population density is largely unknown [4] and will depend on the type of land use, the vulnerability of the vegetation and soils for the different human activities and requirements of the plant and animal species. In lieu of such information, general bench mark values have been used. For their mapping of wilderness areas, Mittermeier et al [40] excluded all areas with a population density of 5 people / km^2^ or above. In their calculations of the human footprint, Sanderson et al [4] assumed that with 10 persons or more per km^2^, human influence could be attributed solely to human population density. This is in line with Gorenflo [41] who found that biodiversity tends to drop off at population densities of more than 10 people per square kilometer. We opted for a more conservative bench mark value of 20 persons / km^2^. This number is based on Kruska et al. [42] who used this number to distinguish between rangeland (< 20 people per km^2^) and the higher impact mixed farming systems (> 20 people / km^2^). Unlike the other estimates, it is not based on specific assumptions on the effect of human population density on biodiversity values. Instead, it is related to conversion of vegetation cover. In rangelands most of the natural vegetation cover is assumed to be maintained, albeit possibly modified due to grazing pressure. In mixed farming systems, an important part of the natural vegetation is assumed to be converted to cropland. We computed human influence scores for densities between 0 and 20 persons per km^2^ to increase linearly from 0 to 100 while the score above 20 persons per km^2^ was held constant at 100. The Afripop data base [22] was used for estimations of the human population densities.

# Sensitivity analysis

A disadvantage of the HI index is that it does not factor in differences in how PVNs are affected by human activities [43]. To describe the human influence as a function of how the different biodiversity components in an PNV are affected by each threat would require substantial parameterization and data currently not available. An alternative approach could be to use expert knowledge [44].

The human influence index depends on the weightings of the individual HI factors. The index is first determined by the percentage of natural vegetation completely converted to croplands, urban areas or roads. For the remaining lands we used an average of the four HI factors, i.e., with equal weights. However, the rescaling of the different factors upon which these indices were based introduced an implicit weighting. For example, if we would use a maximum threshold value of 10 rather than 20 person / km^2^ to compute the HPI, we would also change the relative weight of this factor. To assess how sensitive the HI per PNV is for changes in the weights of the individual factors, we left out the individual human influence factors one by one, after which we recalculated the composite human influence index. Each time we ranked the PNVs again according to their HI scores and compared this to their rankings based on the original HI scores.

Results are given in Table 8, showing changes in ranking ranges from 0 to 12, with an average of 4. For 18 PNVs the largest change in the average HI score is when leaving out the HPI, while removing the ‘percent area crop land’ results for 16 PNVS in the largest change in the average HI score. For the VTI, AI and LPI layers this is the case for 10, 9 and 7 PNVs respectively.

The observed differences in rankings could be because the factors represent different types of human influence with different spatial patterns. An example is the livestock pressure index, which is a relative important determinant of the HI score of the arid vegetation types, but much less so for the HI score of other vegetation types. However, differences can also be related to differences in resolution and accuracy of the data layers used. One of the PNVs affected by such differences is the mangrove, where we see the largest change in rank (12 – if the HPI layer is removed from the equation). A closer examination of the maps shows that none of the individual HI layers cover the whole mangrove distribution area and that there are considerable differences in which areas they cover. This explains most of the change in the average HI scores when removing the HPI layer.

Both examples illustrate that we need to be careful in our interpretation of the results, and it is advisable to always examine the distribution of the individual HI layers (available on http//vegetationmap4africa.org/conservation). They may provide important additional information or help to identify weaknesses in the underlying data layers.

# Tables

**Table 1**. GlobCov land cover types reclassified into one of the two classes of converted lands (A - agriculture and U - urban) or one of the 5 main physiognomic vegetation classes (FO – forest, OF – open forest or woodland, BT – bushland, tickets or wooded grasslands, GH – grasslands or herbaceous vegetation and BS – bare or semi-bare lands). Reclassifications were based on the descriptions of the original land cover classes. Were the original legend expressed the cover of individual LUC types within a compound LUC as a range of percentages, the average of the first LUC was used. Where no percentages were given, we used the following values; 70% for LUC types that covered more than 50% according to the documentation and 30% for LUC types that covered less than 50%.

| **Land use type** | **A** | **U** | **FO** | **OF** | **BT** | **GH** | **BS** |
| --- | --- | --- | --- | --- | --- | --- | --- |
| Post-flooding or irrigated herbaceous crops | 100 | 0 | 0 | 0 | 0 | 0 | 0 |
| Rainfed croplands | 100 | 0 | 0 | 0 | 0 | 0 | 0 |
| Rainfed herbaceous crops | 100 | 0 | 0 | 0 | 0 | 0 | 0 |
| Rainfed shrub or tree crops (cash crops, vineyards, olive tree, orchards…) | 100 | 0 | 0 | 0 | 0 | 0 | 0 |
| Mosaic cropland (50-70%) / vegetation (grassland/shrubland/forest) (20-50%) | 60 | 0 | 40 | 0 | 0 | 0 | 0 |
| Mosaic vegetation (grassland/shrubland/forest) (50-70%) / cropland (20-50%) | 40 | 0 | 20 | 0 | 20 | 20 | 0 |
| Mosaic grassland or shrubland (50-70%) / cropland (20-50%) | 40 | 0 | 0 | 0 | 0 | 60 | 0 |
| Mosaic forest (50-70%) / cropland (20-50%) | 40 | 0 | 60 | 0 | 0 | 0 | 0 |
| Closed to open (>15%) broadleaved evergreen or semi-deciduous forest (>5m) | 0 | 0 | 0 | 100 | 0 | 0 | 0 |
| Closed (>40%) broadleaved evergreen and/or semi-deciduous forest (>5m) | 0 | 0 | 100 | 0 | 0 | 0 | 0 |
| Closed (>40%) broadleaved deciduous forest (>5m) | 0 | 0 | 0 | 100 | 0 | 0 | 0 |
| Open (15-40%) broadleaved deciduous forest/woodland (>5m) | 0 | 0 | 0 | 100 | 0 | 0 | 0 |
| Closed (>40%) needleleaved evergreen forest (>5m) | 0 | 0 | 100 | 0 | 0 | 0 | 0 |
| Open (15-40%) needleleaved deciduous or evergreen forest (>5m) | 0 | 0 | 0 | 100 | 0 | 0 | 0 |
| Closed to open (>15%) mixed broadleaved and needleleaved forest (>5m) | 0 | 0 | 0 | 100 | 0 | 0 | 0 |
| Mosaic forest or shrubland (50-70%) / grassland (20-50%) | 0 | 0 | 0 | 60 | 0 | 40 | 0 |
| Mosaic grassland (50-70%) / forest or shrubland (20-50%) | 0 | 0 | 0 | 40 | 0 | 60 | 0 |
| Closed to open (>15%) (broadleaved or needleleaved, evergreen or deciduous) shrubland (<5m) | 0 | 0 | 0 | 0 | 100 | 0 | 0 |
| Closed to open (>15%) broadleaved deciduous shrubland (<5m) | 0 | 0 | 0 | 0 | 100 | 0 | 0 |
| Closed to open (>15%) herbaceous vegetation (grassland, savannas or lichens/mosses) | 0 | 0 | 0 | 0 | 0 | 100 | 0 |
| Closed (>40%) grassland | 0 | 0 | 0 | 0 | 0 | 100 | 0 |
| Open (15-40%) grassland | 0 | 0 | 0 | 0 | 0 | 100 | 0 |
| Sparse (<15%) vegetation | 0 | 0 | 0 | 0 | 0 | 0 | 100 |
| Sparse (<15%) grassland | 0 | 0 | 0 | 0 | 0 | 0 | 100 |
| Closed to open (>15%) broadleaved forest regularly flooded (semi-permanently or temporarily) - Fresh or brackish water | 0 | 0 | 0 | 100 | 0 | 0 | 0 |
| Closed to open broadleaved forest on (semi-)permanently flooded land - Fresh water | 0 | 0 | 0 | 100 | 0 | 0 | 0 |
| Closed to open broadleaved forest on temporarily flooded land - Fresh water | 0 | 0 | 0 | 100 | 0 | 0 | 0 |
| Closed (>40%) broadleaved forest or shrubland permanently flooded - Saline or brackish water | 0 | 0 | 0 | 100 | 0 | 0 | 0 |
| Closed to open (>15%) grassland or woody vegetation on regularly flooded or waterlogged soil - Fresh, brackish or saline water | 0 | 0 | 0 | 0 | 100 | 0 | 0 |
| Closed to open (>15%) woody vegetation on regularly flooded or waterlogged soil - Fresh or brackish water | 0 | 0 | 0 | 0 | 100 | 0 | 0 |
| Closed to open (>15%) grassland on regularly flooded or waterlogged soil - Fresh or brackish water | 0 | 0 | 0 | 0 | 0 | 100 | 0 |
| Artificial surfaces and associated areas (Urban areas >50%) | 0 | 100 | 0 | 0 | 0 | 0 | 0 |
| Bare areas | 0 | 0 | 0 | 0 | 0 | 0 | 100 |
| Consolidated bare areas (hardpans, gravels, bare rock, stones, boulders) | 0 | 0 | 0 | 0 | 0 | 0 | 100 |
| Non-consolidated bare areas (sandy desert) | 0 | 0 | 0 | 0 | 0 | 0 | 100 |
| Salt hardpans | 0 | 0 | 0 | 0 | 0 | 0 | 100 |
| Permanent snow and ice | 0 | 0 | 0 | 0 | 0 | 0 | 100 |

**Table 2**. GLC2000 land cover types aggregated in 7 land cover or vegetation physiognomic classes. See table 1 for details.

| **Land use type** | **A** | **U** | **FC** | **OF** | **BT** | **GH** | **BS** |
| --- | --- | --- | --- | --- | --- | --- | --- |
| Closed evergreen lowland forest | 0 | 0 | 100 | 0 | 0 | 0 | 0 |
| Degraded evergreen lowland forest | 0 | 0 | 0 | 100 | 0 | 0 | 0 |
| Submontane forest (900 -1500 m) | 0 | 0 | 100 | 0 | 0 | 0 | 0 |
| Montane forest (>1500 m) | 0 | 0 | 100 | 0 | 0 | 0 | 0 |
| Swamp forest | 0 | 0 | 100 | 0 | 0 | 0 | 0 |
| Mangrove | 0 | 0 | 100 | 0 | 0 | 0 | 0 |
| Mosaic Forest / Croplands | 30 | 0 | 70 | 0 | 0 | 0 | 0 |
| Mosaic Forest / Savanna | 0 | 0 | 0 | 100 | 0 | 0 | 0 |
| Closed deciduous forest | 0 | 0 | 100 | 0 | 0 | 0 | 0 |
| Deciduous woodland | 0 | 0 | 0 | 100 | 0 | 0 | 0 |
| Deciduous shrubland with sparse trees | 0 | 0 | 0 | 0 | 100 | 0 | 0 |
| Open deciduous shrubland | 0 | 0 | 0 | 0 | 100 | 0 | 0 |
| Closed grassland^1^ | 0 | 0 | 0 | 0 | 100 | 0 | 0 |
| Open grassland with sparse shrubs^1^ | 0 | 0 | 0 | 0 | 100 | 0 | 0 |
| Open grassland | 0 | 0 | 0 | 0 | 0 | 100 | 0 |
| Sparse grassland | 0 | 0 | 0 | 0 | 0 | 0 | 100 |
| Swamp bushland and grassland | 0 | 0 | 0 | 0 | 100 | 0 | 0 |
| Croplands (>50%) | 70 | 0 | 0 | 30 | 0 | 0 | 0 |
| Croplands with open woody vegetation | 70 | 0 | 0 | 0 | 30 | 0 | 0 |
| Irrigated croplands | 100 | 0 | 0 | 0 | 0 | 0 | 0 |
| Tree crops | 100 | 0 | 0 | 0 | 0 | 0 | 0 |
| Sandy desert and dunes | 0 | 0 | 0 | 0 | 0 | 0 | 100 |
| Stony desert | 0 | 0 | 0 | 0 | 0 | 0 | 100 |
| Bare rock | 0 | 0 | 0 | 0 | 0 | 0 | 100 |
| Salt hardpans | 0 | 0 | 0 | 0 | 0 | 0 | 100 |
| Cities | 0 | 100 | 0 | 0 | 0 | 0 | 0 |

*1 These were grouped with the bushland and wooded grasslands because they almost complete overlap with potential natural vegetation types of that physiognomic group.*

**Table 3**. Classification of the land cover types of the MODIS / IGBP classification scheme in main vegetation physiognomic classes. See Table 1 for more details

| **Land use type** | **A** | **U** | **FC** | **OF** | **BT** | **GH** | **BS** |
| --- | --- | --- | --- | --- | --- | --- | --- |
| Evergreen Needleleaf forest | 0 | 0 | 100 | 0 | 0 | 0 | 0 |
| Evergreen Broadleaf forest | 0 | 0 | 100 | 0 | 0 | 0 | 0 |
| Deciduous Needleleaf forest | 0 | 0 | 0 | 100 | 0 | 0 | 0 |
| Deciduous Broadleaf forest | 0 | 0 | 0 | 100 | 0 | 0 | 0 |
| Mixed forest | 0 | 0 | 100 | 0 | 0 | 0 | 0 |
| Closed shrublands | 0 | 0 | 0 | 0 | 100 | 0 | 0 |
| Open shrublands | 0 | 0 | 0 | 0 | 0 | 0 | 100 |
| Woody savannas | 0 | 0 | 0 | 100 | 0 | 0 | 0 |
| Savannas | 0 | 0 | 0 | 0 | 100 | 0 | 0 |
| Grasslands | 0 | 0 | 0 | 0 | 0 | 100 | 0 |
| Permanent wetlands | 0 | 0 | 0 | 0 | 100 | 0 | 0 |
| Croplands | 100 | 0 | 0 | 0 | 0 | 0 | 0 |
| Urban and built-up | 0 | 100 | 0 | 0 | 0 | 0 | 0 |
| Cropland/Natural vegetation mosaic | 70 | 0 | 0 | 30 | 0 | 0 | 0 |
| Snow and ice | 0 | 0 | 0 | 0 | 0 | 0 | 100 |
| Barren or sparsely vegetated | 0 | 0 | 0 | 0 | 0 | 0 | 100 |

**Table 4**. Reclassification of the Land cover types of the MODIS / University of Maryland (UMD) scheme in main vegetation physiognomic classes. See Table S1 for details.

| **Land use type** | **A** | **U** | **FC** | **OF** | **BT** | **GH** | **BS** |
| --- | --- | --- | --- | --- | --- | --- | --- |
| Evergreen Needleleaf forest | 0 | 0 | 100 | 0 | 0 | 0 | 0 |
| Evergreen Broadleaf forest | 0 | 0 | 100 | 0 | 0 | 0 | 0 |
| Deciduous Needleleaf forest | 0 | 0 | 100 | 0 | 0 | 0 | 0 |
| Deciduous Broadleaf forest | 0 | 0 | 0 | 100 | 0 | 0 | 0 |
| Mixed forest | 0 | 0 | 0 | 100 | 0 | 0 | 0 |
| Closed shrublands | 0 | 0 | 0 | 0 | 100 | 0 | 0 |
| Open shrublands | 0 | 0 | 0 | 0 | 0 | 0 | 100 |
| Woody savannas | 0 | 0 | 0 | 0 | 100 | 0 | 0 |
| Savannas | 0 | 0 | 0 | 0 | 100 | 0 | 0 |
| Grasslands | 0 | 0 | 0 | 0 | 100 | 0 | 0 |
| Croplands | 100 | 0 | 0 | 0 | 0 | 0 | 0 |
| Urban and built-up | 0 | 100 | 0 | 0 | 0 | 0 | 0 |

**Table 5.** Reclassification of road section to travel speed (km/h)

|  | **Travel speed** | |
| --- | --- | --- |
| F_CODE_DES | km/h | min/km |
| Primary and secondary roads | 60 | 1 |
| Bridge/Overpass/Viaduct | 60 | 1 |
| Causeway | 60 | 1 |
| Ford | 40 | 1.5 |
| Trail | 20 | 3 |
| Ferry Crossing | 40 | 1.5 |

**Table 6**. Reclassification of the potential natural vegetation types in main physiognomic classes. See Table 1 for details.

| Land use type | A | U | FC | OF | BT | GH | BS |
| --- | --- | --- | --- | --- | --- | --- | --- |
| Afromontane rain forest (Fa) | 0 | 0 | 100 | 0 | 0 | 0 | 0 |
| Afromontane undifferentiated forest (Fb) | 0 | 0 | 100 | 0 | 0 | 0 | 0 |
| Single-dominant Hagenia abyssinica forest (Fd) | 0 | 0 | 100 | 0 | 0 | 0 | 0 |
| Afromontane moist transitional forest (Fe) | 0 | 0 | 100 | 0 | 0 | 0 | 0 |
| Lake Victoria transitional rain forest (Ff) | 0 | 0 | 100 | 0 | 0 | 0 | 0 |
| Zanzibar-Inhambane transitional rain forest (Fg) | 0 | 0 | 100 | 0 | 0 | 0 | 0 |
| Afromontane dry transitional forest (Fh) | 0 | 0 | 100 | 0 | 0 | 0 | 0 |
| Lake Victoria drier peripheral semi-evergreen Guineo-Congolian rain forest (Fi) | 0 | 0 | 100 | 0 | 0 | 0 | 0 |
| Zambezian dry evergreen forest (Fm) | 0 | 0 | 100 | 0 | 0 | 0 | 0 |
| Zambezian dry deciduous forest and scrub forest (Fn) | 0 | 0 | 100 | 0 | 0 | 0 | 0 |
| Zanzibar-Inhambane lowland rain forest (Fo) | 0 | 0 | 100 | 0 | 0 | 0 | 0 |
| Somalia-Masai Acacia-Commiphora deciduous bushland and thicket (Bd) | 0 | 0 | 0 | 0 | 100 | 0 | 0 |
| Evergreen and semi-evergreen bushland and thicket (Be) | 0 | 0 | 0 | 0 | 100 | 0 | 0 |
| Desert (D) | 0 | 0 | 0 | 0 | 0 | 0 | 100 |
| Climatic grasslands (G) | 0 | 0 | 0 | 0 | 0 | 100 | 0 |
| Somalia-Masai semi-desert grassland and shrubland (S) | 0 | 0 | 0 | 0 | 0 | 0 | 100 |
| Butyrospermum wooded grassland (Wb) | 0 | 0 | 0 | 0 | 100 | 0 | 0 |
| Biotic wooded grassland (We) | 0 | 0 | 0 | 0 | 100 | 0 | 0 |
| Zambezian Kalahari woodland (Wk) | 0 | 0 | 0 | 100 | 0 | 0 | 0 |
| North Zambezian undifferentiated woodland (Wn) | 0 | 0 | 0 | 100 | 0 | 0 | 0 |
| Mopane woodland and scrub woodland (Wo) | 0 | 0 | 0 | 100 | 0 | 0 | 0 |
| Zambezian chipya woodland (Wy) | 0 | 0 | 0 | 100 | 0 | 0 | 0 |
| afromontane bamboo (B) | 0 | 0 | 0 | 0 | 100 | 0 | 0 |
| Montane Ericaceous belt (E) | 0 | 0 | 0 | 0 | 100 | 0 | 0 |
| Lowland bamboo (L) | 0 | 0 | 0 | 0 | 100 | 0 | 0 |
| Mangrove (M) | 0 | 0 | 0 | 100 | 0 | 0 | 0 |
| Palm wooded grassland (P) | 0 | 0 | 0 | 0 | 100 | 0 | 0 |
| Edaphic wooded grassland on drainage-impeded or seasonally flooded soils (wd) | 0 | 0 | 0 | 0 | 100 | 0 | 0 |
| Edaphic grassland on volcanic soils (gv) | 0 | 0 | 0 | 0 | 0 | 100 | 0 |
| Itigi thicket (bi) | 0 | 0 | 0 | 0 | 100 | 0 | 0 |
| Zanzibar-Inhambane coastal mosaic (ZI) | 0 | 0 | 0 | 100 | 0 | 0 | 0 |
| afroalpine vegetation (A) | 0 | 0 | 0 | 0 | 100 | 0 | 0 |
| Afromontane desert (Ad) | 0 | 0 | 0 | 0 | 100 | 0 | 0 |
| Terminalia sericea woodland (Wt) | 0 | 0 | 0 | 100 | 0 | 0 | 0 |
| Vitex-Phyllanthus-Sapium-Terminalia and Terminalia glaucescens woodland (Wv) | 0 | 0 | 0 | 100 | 0 | 0 | 0 |
| Moist combretum wooded grassland (Wcm) | 0 | 0 | 0 | 100 | 0 | 0 | 0 |
| Dry combretum wooded grassland (Wcd) | 0 | 0 | 0 | 100 | 0 | 0 | 0 |
| Wetter miombo woodland (Wmw) | 0 | 0 | 0 | 100 | 0 | 0 | 0 |
| Drier miombo woodland (Wmd) | 0 | 0 | 0 | 100 | 0 | 0 | 0 |
| Miombo woodland on hills and rocky outcrops (Wmr) | 0 | 0 | 0 | 100 | 0 | 0 | 0 |
| Acacia-Commiphora stunted bushland (Bds) | 0 | 0 | 0 | 0 | 0 | 0 | 100 |
| Acacia-Commiphora deciduous wooded grassland (Bdw) | 0 | 0 | 0 | 0 | 0 | 100 | 0 |
| Catena of North Zambezian Undifferentiated woodland and edaphic grassland on drainage-impeded or seasonally flooded soils (Wn/g) | 0 | 0 | 0 | 0 | 100 | 0 | 0 |
| Transitional zone of drier miombo woodland and North Zambezian Undifferentiated woodland (Wmd/Wn) | 0 | 0 | 0 | 0 | 100 | 0 | 0 |
| Mosaic of Montane Ericaceous belt and Single-dominant Widdringtonia whytei forest (E/Fc)^2^ | 0 | 0 | 0 | 0 | 100 | 0 | 0 |
| Edaphic grassland on drainage-impeded, seasonally flooded soils or freshwater swamp (g/X) | 0 | 0 | 0 | 0 | 0 | 100 | 0 |
| Zambezian Kalahari woodlands within edaphic grassland on drainage-impeded or seasonally flooded soils (Wk/g) | 0 | 0 | 0 | 0 | 100 | 0 | 0 |
| Bush groups, typically around termitaria, within grassy drainage zones (T/g) | 0 | 0 | 0 | 0 | 100 | 0 | 0 |
| Transitional zone of drier miombo woodland and Somalia-Masai Acacia-Commiphora deciduous bushland and thicket (Wmd/Bd) | 0 | 0 | 0 | 0 | 100 | 0 | 0 |
| Catena of Acacia-Commiphora deciduous wooded grassland, Combretum wooded grassland and edaphic grassland on drainage-impeded or seasonally flooded soils (Bdw/Wc/g | 0 | 0 | 0 | 0 | 100 | 0 | 0 |
| Afromontane forest – grasslands mosaic^1^ | 0 | 0 | 0 | 0 | 0 | 100 | 0 |

*1 There is no conclusive evidence about the spatial configuration of the grassland-forest mosaic within this mapping unit. The mosaic was therefore reclassified as 'Grasslands / herbaceous. This may results in an underestimation of the extent of deforestation within these areas.*

*2 Mosaic of Montane Ericaceous belt and Single-dominant Widdringtonia whytei forest was grouped with the bushlands as there is no information about the spatial configuration of the two components. This is likely to results in an underestimation of the extent of the changes in forest cover*.

**Table 7**. Conversion of land use classes from the GLC 2000 land cover layer to travel speed (km/h).

| **Map** | **GLC 2000** | |
| --- | --- | --- |
| **ID** | **Regional classification** | **(mins / km)** |
| 1 | Closed evergreen lowland forest | 60 |
| 2 | Degraded evergreen lowland forest | 60 |
| 3 | Submontane forest (900 -1500 m) | 60 |
| 4 | Montane forest (>1500 m) | 60 |
| 5 | Swamp forest | 60 |
| 6 | Mangrove | 60 |
| 7 | Mosaic Forest / Croplands | 36 |
| 8 | Mosaic Forest / Savanna | 48 |
| 9 | Closed deciduous forest | 60 |
| 10 | Deciduous woodland | 48 |
| 11 | Deciduous shrubland with sparse trees | 36 |
| 12 | Open deciduous shrubland | 36 |
| 13 | Closed grassland | 36 |
| 14 | Open grassland with sparse shrubs | 24 |
| 15 | Open grassland | 24 |
| 16 | Sparse grassland | 24 |
| 17 | Swamp bushland and grassland | 60 |
| 18 | Croplands (>50%) | 36 |
| 19 | Croplands with open woody vegetation | 36 |
| 20 | Irrigated croplands | 36 |
| 21 | Tree crops | 36 |
| 22 | Sandy desert and dunes | 24 |
| 23 | Stony desert | 24 |
| 24 | Bare rock | 24 |
| 25 | Salt hardpans | 24 |
| 26 | Waterbodies | 60 |
| 27 | Cities | 2 |

Table 8. Rank order of PNVs according to the average human influence score (HI) within their distribution area, and the changes in the rank order after leaving out one by one the individual human influence factors AI (accessibility index), crops (percent area converted to crop lands), HPI (human population index), LPI (livestock pressure index) and VTI (vegetation transformation index). The last column gives the maximum absolute change across these five factors.

|  |  | **Difference with original HI** | | | | | |
| --- | --- | --- | --- | --- | --- | --- | --- |
| PNV | HI | - AI | - crops | - HPI | - LPI | -VTI | Max. |
| Fa | 45 | 0 | 0 | 1 | 0 | 2 | 2 |
| Fb | 39 | -1 | 0 | 1 | -1 | 2 | 2 |
| Fd | 22 | -6 | 9 | -3 | 0 | 1 | 9 |
| Fe | 49 | 2 | 0 | 1 | 1 | 0 | 2 |
| Ff | 50 | 0 | 0 | 0 | 0 | 0 | 0 |
| Fg | 28 | -6 | 0 | 5 | -2 | 3 | -6 |
| Fh | 46 | 0 | -2 | 0 | 0 | 0 | -2 |
| Fi | 47 | -1 | 0 | 0 | -2 | 0 | -2 |
| Fm | 7 | -1 | -1 | 0 | -2 | 4 | 4 |
| Fn | 19 | -2 | -3 | -5 | -2 | 6 | 6 |
| Fo | 35 | 0 | -2 | -1 | 0 | 0 | -2 |
| CM | 34 | 3 | 1 | -1 | 0 | 2 | 3 |
| M | 25 | -2 | -5 | 12 | -4 | -2 | 12 |
| Wcd | 42 | 0 | 2 | 0 | 0 | 2 | 2 |
| Wcm | 48 | -1 | 2 | -1 | 1 | 0 | 2 |
| Wk | 14 | 1 | -1 | 0 | -1 | 2 | 2 |
| Wmd | 17 | 0 | -1 | -1 | 0 | 1 | -1 |
| Wmr | 36 | 0 | 2 | 2 | 0 | 0 | 2 |
| Wmw | 16 | 0 | -1 | 7 | 0 | -1 | 7 |
| Wn | 32 | 3 | 0 | 4 | -1 | 1 | 4 |
| Wo | 12 | -3 | 5 | 0 | -1 | 4 | 5 |
| Wv | 21 | -2 | 9 | -1 | 2 | 1 | 9 |
| Wy | 11 | 0 | -5 | 3 | -3 | -3 | -5 |
| Wmd/Wn | 4 | 2 | 2 | -2 | -1 | 0 | -2 |
| Bd | 26 | 4 | -3 | -5 | 1 | 0 | -5 |
| Bds | 15 | 5 | -4 | -4 | 5 | -3 | 5 |
| Bdw | 40 | 1 | -1 | 0 | 3 | -1 | 3 |
| Bdw/Wc/g | 29 | 4 | -2 | -3 | 2 | -1 | 4 |
| Be | 41 | 0 | -3 | 0 | 0 | -1 | -3 |
| bi | 23 | 4 | -2 | -6 | 0 | -1 | -6 |
| L | 37 | -1 | 2 | 0 | -1 | -2 | -2 |
| P | 43 | 0 | 0 | -2 | -1 | -1 | -2 |
| T/g | 9 | 0 | 0 | 4 | -2 | 0 | 4 |
| Wb | 44 | 0 | 2 | 1 | 1 | -1 | 2 |
| wd | 38 | 1 | 0 | -1 | -1 | 0 | -1 |
| We | 33 | 3 | -3 | 0 | 1 | -1 | -3 |
| Wk/g | 1 | 0 | 0 | -2 | -2 | -1 | -2 |
| Wmd/Bd | 27 | 1 | 1 | -3 | 1 | -1 | -3 |
| Wn/g | 20 | 0 | 0 | -1 | 0 | -2 | -2 |
| A | 2 | -3 | -1 | 0 | 1 | 1 | -3 |
| Ad | 5 | -2 | 1 | 4 | -2 | 0 | 4 |
| B | 31 | -2 | 7 | 4 | 3 | 2 | 7 |
| E | 6 | -6 | 1 | 2 | -2 | 0 | -6 |
| E/Fc | 3 | -1 | -3 | -7 | -3 | -4 | -7 |
| D | 10 | 4 | -1 | -6 | 8 | -1 | 8 |
| S | 8 | 5 | -2 | -3 | 4 | -2 | 5 |
| G | 24 | 0 | 1 | -2 | 0 | 1 | -2 |
| g/X | 30 | -2 | 3 | 10 | -1 | -3 | 10 |
| gm/F | 13 | -1 | -1 | -2 | 1 | -2 | -2 |
| gv | 18 | 0 | -3 | 1 | 0 | -1 | -3 |

# References

1. Fritz S, McCallum I, Schill C, Perger C, Grillmayer R, Achard F, et al. Geo-Wiki.Org: The Use of Crowdsourcing to Improve Global Land Cover. Remote Sens. 2009;1: 345–354. doi:10.3390/rs1030345

2. Fritz S, McCallum I, Schill C, Perger C, See L, Schepaschenko D, et al. Geo-Wiki: An online platform for improving global land cover. Environ Model Softw. 2012;31: 110–123. doi:10.1016/j.envsoft.2011.11.015

3. Schneider A, Friedl MA, Potere D. A new map of global urban extent from MODIS satellite data. Environ Res Lett. 2009;4: 044003. doi:10.1088/1748-9326/4/4/044003

4. Sanderson EW, Jaiteh M, Levy MA, Redford KH, Wannebo AV, Woolmer G. The human footprint and the last of the wild. BioScience. 2002;52: 891–904.

5. Defourny P, Bontemps S, Obsomer V, Schouten L, Bartalev S, Herold M, et al. Accuracy assessment of global land cover maps: lessons learnt from the GlobCover and GlobCorine experiences. ESA; 2010.

6. Fritz S, See L. Identifying and quantifying uncertainty and spatial disagreement in the comparison of Global Land Cover for different applications. Glob Change Biol. 2008;14: 1057–1075.

7. Fritz S, See L, Rembold F. Comparison of global and regional land cover maps with statistical information for the agricultural domain in Africa. Int J Remote Sens. 2010;31: 2237–2256.

8. Giri C, Zhu Z, Reed B. A comparative analysis of the Global Land Cover 2000 and MODIS land cover data sets. Remote Sens Environ. 2005;94: 123–132.

9. Hansen MC, Reed B. A comparison of the IGBP DISCover and University of Maryland 1 km global land cover products. Int J Remote Sens. 2000;21: 1365–1373. doi:10.1080/014311600210218

10. Vancutsem C, Marinho E, Kayitakire F, See L, Fritz S. Harmonizing and Combining Existing Land Cover/Land Use Datasets for Cropland Area Monitoring at the African Continental Scale. Remote Sens. 2012;5: 19–41. doi:10.3390/rs5010019

11. Bontemps S, Defourny P, Van Bogaert E. GLOBCOVER 2009 Products Description and Validation Report [Internet]. ESA 2010 and UCLouvain; 2010 p. 30. Available: http://ionia1.esrin.esa.int/

12. ESA. GlobCover 2005-06 (version 2.2) [Internet]. 2.2 ed. ESA GlobCover Project, led by MEDIAS-France; 2007. Available: http://postel.mediasfrance.org

13. JRC. Global Land Cover 2000 database version 3 [Internet]. 3rd ed. European Commission, Joint Research Centre; 2003. Available: http://bioval.jrc.ec.europa.eu/products/glc2000/glc2000.php

14. LP DAAC. MODIS Land Cover Type Yearly L3 Global 500 m SIN Grid (MCD12Q1) [Internet]. Sioux Falls: NASA Land Processes Distributed Active Archive Center (LP DAAC), U.S. Geological Survey (USGS) Earth Resources Observation and Science (EROS) Center; 2009. Available: https://lpdaac.usgs.gov/lpdaac/products/modis_products_table/land_cover/yearly_l3_global_500_m/mcd12q1

15. Friedl MA, McIver DK, Hodges JCF, Zhang XY, Muchoney D, Strahler AH, et al. Global land cover mapping from MODIS: algorithms and early results. Remote Sens Environ. 2002;83: 287–302.

16. Verburg PH, Ellis EC, Letourneau A. A global assessment of market accessibility and market influence for global environmental change studies. Environ Res Lett. 2011;6: 034019. doi:10.1088/1748-9326/6/3/034019

17. GRASS Development Team. Geographic Resources Analysis Support System (GRASS GIS) Software, version 7.0 [Internet]. USA: Open Source Geospatial Foundation; 2014. Available: http://grass.osgeo.org

18. Nelson A. Travel time to major cities: A global map of accessibilty [Internet]. Ispra Italy: Global Environment Monitoring Unit - Joint Research Centre of the European Commission; 2008. Available: http://bioval.jrc.ec.europa.eu/products/gam/index.htm

19. Wagtendonk JW van., Benedict JM. Travel time variation on backcountry trails. J Leis Res. 1980;12: 100–106.

20. Moore ID, Grayson RB, Ladson AR. Digital terrain modelling. A review of hydrological, geomorphological, and biological applications. Hydrol Process. 1991;5: 3–30.

21. De Wit M, Stankiewicz J. Changes in Surface Water Supply Across Africa with Predicted Climate Change. Science. 2006;311: 1917–1921.

22. Linard C, Gilbert M, Snow RW, Noor AM, Tatem AJ. Population Distribution, Settlement Patterns and Accessibility across Africa in 2010. PLoS ONE. 2012;7: e31743. doi:10.1371/journal.pone.0031743

23. Leeuw PN de, Reid R. Impact of human activities and livestock on the African environment: an attempt to partition the pressure. In: Wilson RT, Ehui S, Simon Mack, editors. Livestock development strategies for low income countries - Proceedings of the joint FAO/ILRI roundtable on livestock development strategies for low income countries. Rome, Nairobi: Food and Agriculture Organization (FAO), International Livestock Research Institute (ILRI); 1995. Available: http://www.ilri.cgiar.org/InfoServ/Webpub/fulldocs/X5462E/x5462e00.htm

24. Ahmed AGM, Azeze A, Babiker M, Tsegaye D. Post-drought recovery strategies among the pastoral households in the horn ofafrica: a review. Organization for Social Science Research in Eastern and Southern Africa (OSSREA); 2003.

25. Gryseels G. Role of livestock on mixed smallholder farms in the Ethiopian highlands. A case study from the Baso and Worena Wereda near Debre Berhan. Ph.D., Agricultural University, Wageningen, the Netherlands. 1988.

26. De Leeuw PN, Rey B. Analysis of current trends in the distribution patterns of ruminant livestock in tropical Africa. World Anim Rev. 1995;83: 47–59.

27. Wint GRW, Robinson TP. Gridded livestock of the world 2007 [Internet]. Rome: Food and Agricultural Organization of the United Nations; 2007. Available: http://www.fao.org/ag/AGAinfo/resources/en/glw/default.html

28. Boudet G. Problems encountered in estimating the rate of stocking of “natural pastureland” in a tropical zone. Proceedings of the seminar Evaluation and mapping of tropical African rangelands Bamako, Mali 3-8 March 1975. INTERNATIONAL LIVESTOCK CENTRE FOR AFRICA; 1975. Available: http://www.fao.org/wairdocs/ilri/x5543b/x5543b14.htm

29. Peden D, Tadesse G, Misra AK, Comprehensive Assessment of Water Management in Agriculture. Water and livestock for human development. Water for Food, Water for Life A Comprehensive Assessment of Water Management in Agriculture. IWMI; 2007. pp. 485–514.

30. Herrero M, Thornton PK, Nicholson CF, Blummel M. A framework to assess the impacts of feed resource interventions in smallholder systems. System Wide Livestock Programme, ILRI; 2005 p. 28.

31. Rutagwenda T, Wanyoike MM. Seasonal changes in livestock diets in a semi-arid environment in northern Kenya. In: Stares JES, Said AN, Kategile JA, editors. The Complementarity of Feed Resources for Animal Production in Africa. Addis Ababa, Ethiopia: African Feeds Research Network. ILCA (International Livestock Centre for Africa); 1992. Available: http://www.ilri.org/InfoServ/Webpub/Fulldocs/X5519b/x5519b09.htm#seasonal%20changes%20in%20livestock%20diets%20in%20a%20semi%20arid%20environment%20in%20northern%20kenya

32. Van Breugel P, Herrero M, van de Steeg J, Peden D. Livestock Water Use and Productivity in the Nile Basin. Ecosystems. 2010;13: 205–221. doi:10.1007/s10021-009-9311-z

33. VITO. Dry Matter Productivity [Internet]. European Community, Directorate General of the Joint Research Center (JRC), Institute for Protection and Security of the Citizen (IPSC), AGRIFISH Unit (MARS-FOOD project ); 2012. Available: http://www.vgt4africa.org/

34. Fratkin EM, Roth EA. As Pastoralists Settle · Social, Health, and Economic Consequences of the Pastoral Sedentarization in Marsabit District, Kenya. Berlin: Springer; 2005.

35. Holechek JL, Gomez H, Molinar F, Galt D. Grazing Studies: What We’ve Learned. Rangelands. 1999;21: 12–16.

36. De Leeuw PN, Tothill JC. The Concept of Rangeland Carrying Capacity in Sub-Saharan Africa: Myth Or Reality. Overseas Development Institute, Pastoral Development Network; 1990.

37. Trabucco A, Zomer RJ. Global Aridity Index (Global-Aridity) and Global Potential Evapo-Transpiration (Global-PET) Geospatial Database [Internet]. Published online, available from the CGIAR-CSI GeoPortal: CGIAR Consortium for Spatial Information (CGIAR-CSI); 2009. Available: http://www.csi.cgiar.org

38. Zomer RJ, Trabucco A, Bossio DA, Verchot LV. Climate change mitigation: A spatial analysis of global land suitability for clean development mechanism afforestation and reforestation. Agric Ecosyst Environ. 2008;126: 67–80.

39. Cincotta RP, Wisnewski J, Engelman R. Human population in the biodiversity hotspots. Nature. 2000;404: 990–992.

40. Mittermeier RA, Mittermeier CG, Brooks TM, Pilgrim JD, Konstant WR, da Fonseca GAB, et al. Wilderness and biodiversity conservation. Proc Natl Acad Sci U S A. 2003;100: 10309–10313. doi:10.1073/pnas.1732458100

41. Gorenflo LJ. Human Demography and Conservation in the Apache Highlands Ecoregion, US–Mexico Borderlands. In: Cincotta RP, Gorenflo LJ, editors. Human Population - Its Influences on Biological Diversity. Springer; 2011. pp. 153–176. Available: http://www.springer.com.ep.fjernadgang.kb.dk/life+sciences/ecology/book/978-3-642-16706-5

42. Kruska RL, Reid RS, Thornton PK, Henninger N, Kristjanson PM. Mapping livestock-oriented agricultural production systems for the developing world. Agric Syst. 2003;77: 39–63.

43. Sanderson EW, Redford KH, Vedder A, Coppolillo PB, Ward SE. A conceptual model for conservation planning based on landscape species requirements. Landsc Urban Plan. 2002;58: 41–56. doi:10.1016/S0169-2046(01)00231-6

44. Jarvis A, Touval JL, Schmitz MC, Sotomayor L, Hyman GG. Assessment of threats to ecosystems in South America. J Nat Conserv. 2010;18: 180–188. doi:10.1016/j.jnc.2009.08.003
