# Supplementary figures and images for: Environmental Gap Analysis to Prioritize Conservation Efforts in Eastern Africa
Source: PLoS One. 2015 Apr 9;10(4):e0121444. doi: 10.1371/journal.pone.0121444 (PMC4391866; doi:10.1371/journal.pone.0121444)

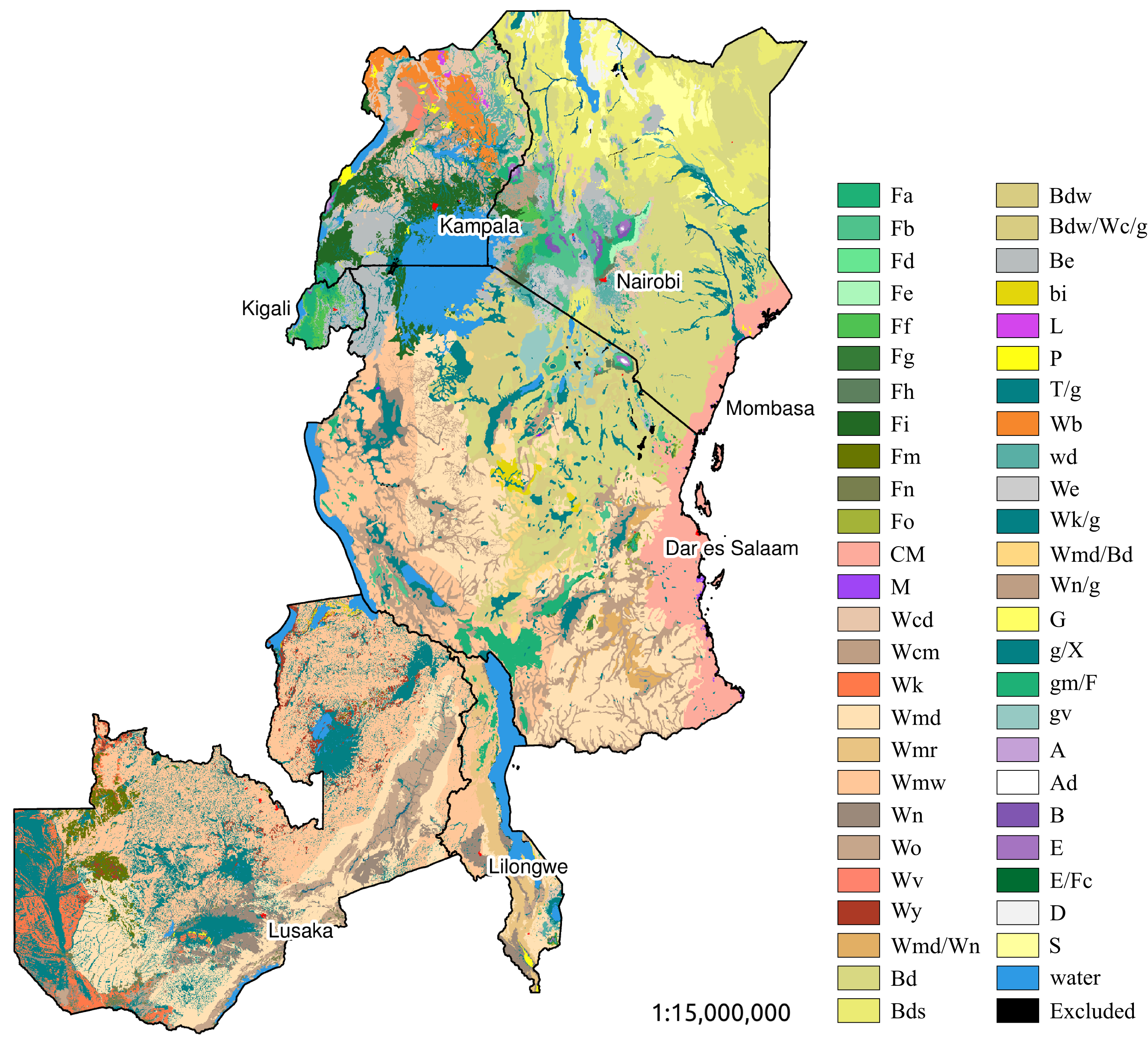

Supplement: S1 Fig — Potential natural vegetation (PNV) map based on the VECEA PNV map by van Breugel et al. [62]. The full names of the potential natural vegetation types, corresponding to the codes in the legend, are provided in Table 1. PNVs marked with an asterisk were not used in our analysis. For reference purposes the position of capital cities are indicated, with their extent based on the MODIS 2009 urban areas mask [75]. (TIF) [file pone.0121444.s003.tif]

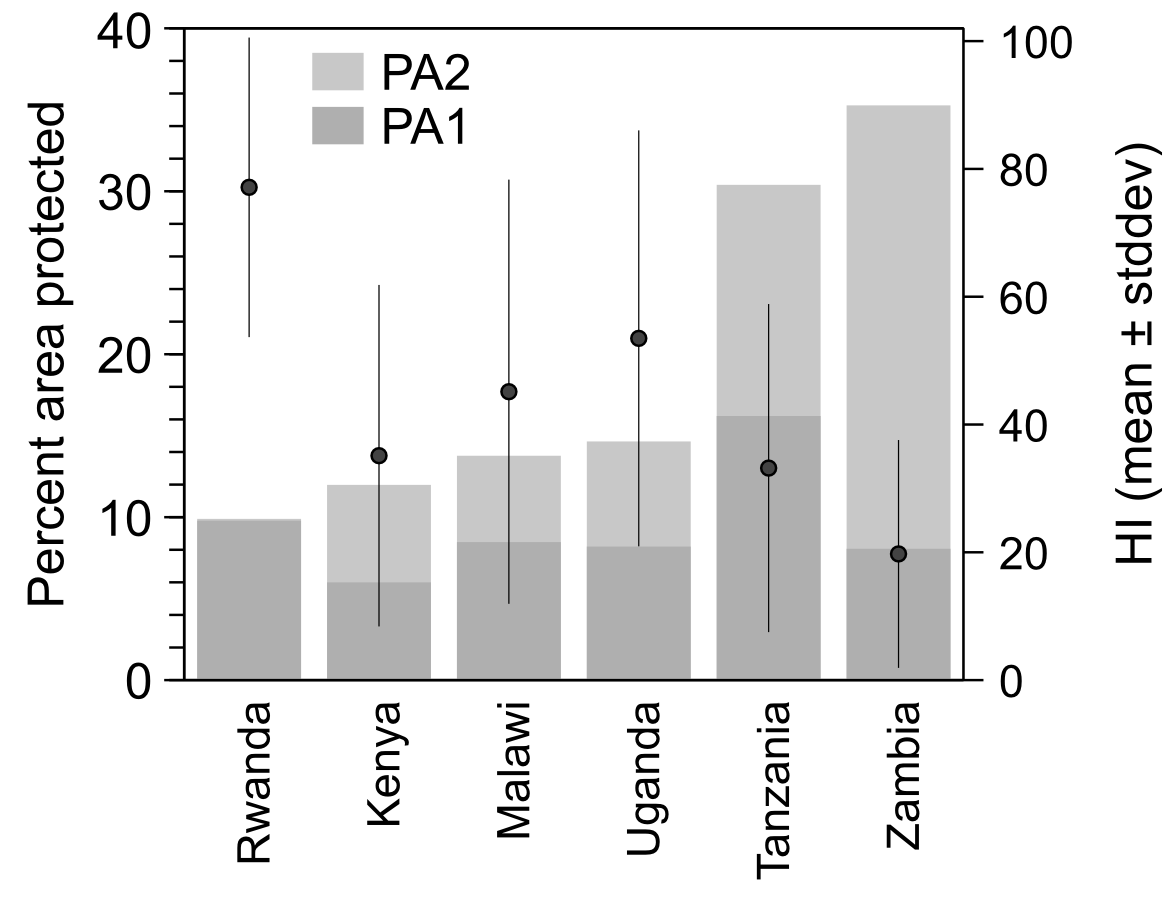

Supplement: S2 Fig — Stacked barplot with with the percent area protected by country within the PA1 (IUCN categories Ib, II, III and IV) and PA2 (IUCN category VI and unclassified) protected areas. The dots and error bars give the average and standard deviation of the human influence by country. (TIF) [file pone.0121444.s004.tif]
